# Cytokine release and gastrointestinal symptoms after gluten challenge in celiac disease

**DOI:** 10.1126/sciadv.aaw7756

**Published:** 2019-08-07

**Authors:** Gautam Goel, Jason A. Tye-Din, Shuo-Wang Qiao, Amy K. Russell, Toufic Mayassi, Cezary Ciszewski, Vikas K. Sarna, Suyue Wang, Kaela E. Goldstein, John L. Dzuris, Leslie J. Williams, Ramnik J. Xavier, Knut E. A. Lundin, Bana Jabri, Ludvig M. Sollid, Robert P. Anderson

**Affiliations:** 1 Division of Gastroenterology and Center for Computational and Integrative Biology, Massachusetts General Hospital, Boston, MA, USA.; 2 Immunology Division, The Walter and Eliza Hall Institute, Parkville, VIC, Australia.; 3 Department of Medical Biology, University of Melbourne, Parkville, VIC, Australia.; 4 Department of Gastroenterology, The Royal Melbourne Hospital, Parkville, VIC, Australia.; 5 Centre for Food and Allergy Research, Murdoch Children’s Research Institute, Parkville, VIC, Australia.; 6 Department of Immunology and KG Jebsen Coeliac Disease Research Centre, University of Oslo and Oslo University Hospital-Rikshospitalet, Oslo, Norway.; 7 Department of Pediatrics, Department of Medicine, University of Chicago, Chicago, IL, USA.; 8 ImmusanT Inc., Cambridge, MA, USA.; 9 Department of Gastroenterology and KG Jebsen Coeliac Disease Research Centre, University of Oslo and Oslo University Hospital-Rikshospitalet, Oslo, Norway.

## Abstract

Celiac disease (CeD), caused by immune reactions to cereal gluten, is treated with gluten -elimination diets. Within hours of gluten exposure, either perorally or extraorally by intradermal injection, treated patients experience gastrointestinal symptoms. To test whether gluten exposure leads to systemic cytokine production time -related to symptoms, series of multiplex cytokine measurements were obtained in CeD patients after gluten challenge. Peptide injection elevated at least 15 plasma cytokines, with IL-2, IL-8, and IL-10 being most prominent (fold-change increase at 4 hours of 272, 11, and 1.2, respectively). IL-2 and IL-8 were the only cytokines elevated at 2 hours, preceding onset of symptoms. After gluten ingestion, IL-2 was the earliest and most prominent cytokine (15-fold change at 4 hours). Supported by studies of patient-derived gluten-specific T cell clones and primary lymphocytes, our observations indicate that gluten-specific CD4^+^ T cells are rapidly reactivated by antigen -exposure likely causing CeD-associated gastrointestinal symptoms.

## Teaser

Acute gastrointestinal symptoms induced by gluten, within 4 hours, are mediated by reactivation of antigen-specific memory T cells.

## INTRODUCTION

Celiac disease (CeD) is a prevalent autoimmune disorder caused by ingested cereal gluten proteins (*1*). CeD is hallmarked by an acquired immune response to gluten, and the only available treatment for the disease is a lifelong gluten-free diet (GFD). The immune response to gluten in CeD is driven by CD4^+^ T cells specific for deamidated gluten peptides that uniquely bind to disease-associated human leukocyte antigen (HLA)–DQ allotypes (*2*).

If treated CeD patients, i.e., those following a strict GFD, are exposed to gluten-containing food, they typically suffer from gastrointestinal reactions occurring 1 to 2 hours after the gluten exposure (*3*). There is currently no explanation for the acute gluten-induced symptoms seen in treated CeD patients. Gastrointestinal manifestations similar to severe gluten exposure symptoms in CeD can be prominent in the cytokine release syndrome after the first infusion of biologics that activate T cells (*4*). Although gluten-stimulated cytokine profiles of mucosal tissue and gluten-specific CD4^+^ T cells in long-term culture have been extensively investigated (*2*), the symptoms of acute gluten exposure in CeD have not been clinically linked to cytokine changes (*5*).

Interest in cytokine release following reactivation of gluten immunity was prompted by studies in CeD patients that assessed the clinical and immunological effects of an investigational antigen-specific immunotherapy (Nexvax2; ImmusanT, Cambridge, MA, USA) (*6*). Nexvax2 is an equimolar mixture of three soluble peptides that were reported by Tye-Din *et al*. (*7*) and correspond to 15- or 16-mer peptide fragments of native gluten proteins with glutamate residues replacing glutamine at sites predicted to be susceptible to deamidation by transglutaminase 2 (TG2). CD4^+^ T cells specific for the overlapping HLA-DQ2.5–restricted epitopes in these “gluten peptides” (DQ2·5-glia-α1a/α2, DQ2·5-glia-ω1/ω2, and DQ2.5-hor-3/var, DQ2·5-glia-γ5) account for the majority of peripheral blood and intestinal T cells responding to gluten, hordein (barley), and secalin (rye) (*7*, *8*). Immunodominant B cell epitopes are also represented in Nexvax2 gluten peptides (*9*), but CeD patients receiving Nexvax2 show no complement activation or induction of anti-Nexvax2 antibody (*6*, *10*). Nexvax2 peptides have no known effects on innate immune cells.

Two separate phase 1 double-blind, placebo-controlled, ascending intradermal dose studies of Nexvax2 were completed in HLA-DQ2.5^+^ CeD patients on GFD and have been described in detail elsewhere (*6*). The designs and readouts of the two studies were closely aligned except for the dose regimen. In these studies, nausea, vomiting, and abdominal pain frequently followed within 2 to 5 hours after the first dose of gluten peptides, which determined the maximum tolerated dose (150 μg). Injection site reactions resembling a cutaneous response to recall antigen (*11*) were not observed. We now report plasma cytokine profiles and how they correlated with clinical assessments after the first and last doses of Nexvax2 gluten peptides. Our observations from these phase 1 studies led us to hypothesize that cytokine release occurs following natural gluten exposure and could be used to implicate which arms of the immune system drive early symptoms. The aim of the present study was to characterize systemic cytokine profiles and their relation to acute symptoms in CeD patients after reactivation of gluten immunity, either by injection of synthetic gluten peptides or by feeding of natural gluten.

## RESULTS

### Multiplex evaluation of plasma cytokines after first intradermal injection of gluten peptides

**Fig. 1:**
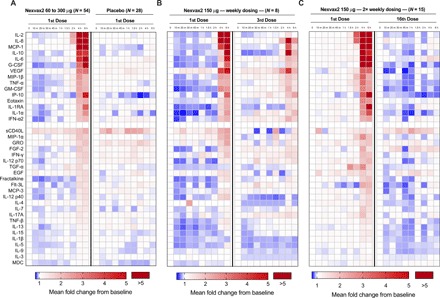
Activation of immune response by gluten-derived immunodominant peptides. (**A**) Heatmap of average baseline-adjusted fold change response in groups of patients either treated with first dose of Nexvax2 (60, 90, 150, or 300 μg) or matched placebo control. Only IL-2 and IL-8 showed significant elevations as early as 2 hours. Temporal response typically peaked at 4 hours except for some cytokines, such as IP-10 and G-CSF, which peaked at 6 hours after dose. (**B**) Heatmap of average baseline-adjusted fold change response in groups of patients either treated with first dose of Nexvax2 (150 μg) or third weekly dose. (**C**) Heatmap of average baseline-adjusted fold-change response in groups of patients either treated with first dose of Nexvax2 (150 μg) or 16th twice-weekly dose. VEGF, vascular endothelial growth factor.

Plasma samples were collected at 10 time points up to 6 hours after the first dose of study drug in two phase 1 studies. Stored plasma from all 54 patients receiving gluten peptides, and all 28 patients receiving placebo (0.9% sodium chloride) were assessed for 38 cytokines and chemokines using a multiplex magnetic bead assay. A highly consistent temporal cytokine profile was observed starting at 2 hours after dose in participants who received gluten peptides (Fig. 1A and table S1). Together, 15 cytokines and chemokines showed statistically significant elevations by 6 hours. Highest median peak elevations, which were assessed as fold change from baseline, were for monocyte chemoattractant protein 1 (MCP-1/CCL2), interleukin-8 (IL-8; CXCL8), and IL-2.

With onset at 2 hours, IL-2 and IL-8 were the first cytokines to become significantly elevated and, along with macrophage inflammatory protein (MIP)–1β and tumor necrosis factor–α (TNF-α), were also the first to reach peak plasma levels (4 hours). Other cytokines plateaued from 4 hours (e.g., IL-10) or were highest at 6 hours [e.g. interferon- (IFN-)-inducible polypeptide-10 (IP-10), which is C-X-C motif chemokine (CXCL)10] and may have peaked later than 6 hours] and may have peaked later than 6 hours. Plasma elevations of IFN-γ and other T cell–derived cytokines such as IL-4, IL-5, IL-13, and IL-17 were not evident, but increased plasma levels of IP-10 potentially reflected increased tissue production of IFN-γ (*12*). MDC/CCL22 (macrophage-derived chemokine/C-C motif chemokine 22), a chemokine released by activated dendritic cells (DCs) and B cells, was also not evident (*13*). Soluble CD40 ligand (sCD40L) and the chemokine, GRO/CXCL1 (growth-regulated oncogene/CXCL1), showed elevated plasma levels from 10 min in both placebo-treated and gluten peptide–treated patients, which is likely to be an artifact related to release by activated platelets (*14*, *15*).

A second multiplex assay was used to replicate and more broadly define the plasma cytokine signature associated with administration of gluten peptides. A 92-plex proximity extension assay (PEA) was used to reassess plasma samples collected at 2, 4, and 6 hours after dose from six patients, four had been administered with 150 μg of Nexvax2 and two had received placebo. For the cytokines that showed greatest increases in the magnetic bead assay, fold changes from baseline measured by the two assays were highly correlated (MCP-1: Pearson *r* = 0.962, *P* < 0.0001; IL-8: *r* = 0.941, *P* < 0.0001; IL-2: *r* = 0.960, *P* < 0.0001; and IL-10: *r* = 0.983, *P* < 0.0001). Additional cytokines and chemokines that were not included in the original 38-plex panel were also prominent. In particular, relative elevations in MIP-3α/CCL20, a chemotactic factor for effector/memory T cells and B cells and immature DCs at skin and mucosal surfaces (*16*), were similar to IL-8, elevations in CXCL9 and IL-17C were similar to IP-10, and increases in MCP-1 were similar to MCP-2, matrix metalloproteinase 1, and oncostatin, a cytokine that promotes intestinal inflammation (fig. S1) (*17*). A trend for plasma elevations of IFN-γ was observed at 6 hours; however, stronger evidence for induction of IFN-γ after administration of gluten peptides was provided by the finding of elevations in downstream targets of IFN-γ, namely, CXCL9 and CXCL11, in addition to IP-10. These analyses indicate that the antigenic potency of short, soluble gluten peptides identified in vitro as HLA-DQ2.5–restricted gluten epitopes can be confirmed in HLA-DQ2.5^+^ CeD patients by measuring elevations in plasma cytokines from 2 to 6 hours after intradermal injection.

### Reevaluation of plasma cytokines by electrochemiluminescence assay

**Fig. 2:**
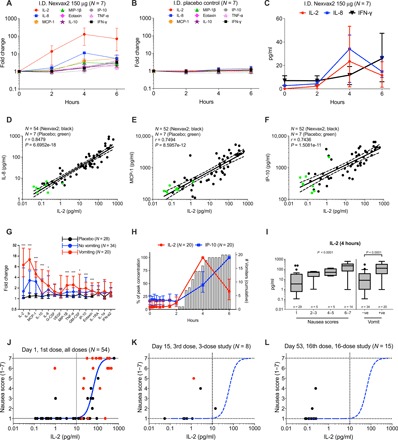
Assessment of select immune response by gluten peptides using a sensitive multiplex assay. (**A**) Baseline-adjusted fold-change response assessed in 150 μg of Nexvax2-treated cohort. Median and IQRs are shown. (**B**) Responses in placebo-treated patients. (**C**) Differences in activation response of IL-2, IL-8, and IFN-γ as judged by cytokine concentrations. (**D** to **F**) Pearson’s correlation analysis of IL-2 concentration at 4 hours after dose with IL-8 at 4 hours (D), MCP-1 at 4 hours (E), and IP-10 at 6 hours after dose (F) are shown. Green dots indicate cytokine response in placebo-treated patients (*n* = 7). (**G**) Baseline-adjusted fold-change response at onset of vomiting in Nexvax2- and placebo-treated patients. Median values and IQRs are shown. Response in participants who vomited was compared to placebo response using a Mann-Whitney *U* test. Significant cytokines are indicated with asterisks (***P < 0.001; *P < 0.05). (**H**) Kinetics of cytokine elevation (on left *y* axis) overlaid on incidence of vomiting (on right *y* axis). Concentration profiles were normalized by peak concentration value and expressed as a percentage. Median values and IQRs are shown. (**I**) IL-2 concentration stratified by either patient-reported nausea score or occurrence of vomiting is shown. For nausea scores, a *P* value was estimated by Kruskal-Wallis test. For vomiters and nonvomiters, a *P* value was computed by Mann-Whitney *U* test, and significance was further confirmed by regression modeling. (**J**) A sigmoidal dose-response relationship is observed between levels of plasma IL-2 and magnitude of self-reported nausea score after first dose of Nexvax2 (60, 90, 150, or 300 μg). Blue line represents a 4-parameter logistic dose-response curve. Red dot indicates that patient vomited after receiving Nexvax2. Significant occurrence of high-grade nausea and vomiting are observed at IL-2 > 10 pg/ml (model-based threshold estimate). Intensity of IL-2 induction and self-reported attenuated scores are attenuated after third weekly dose (**K**) and absent after 16th twice-weekly dose (**L**).

The measurement of IL-2 and IL-8 release after administering gluten peptides was compromised by baseline plasma concentrations of these cytokines frequently being below the lower limit of detection for both the magnetic bead assay and also the PEA. The electrochemiluminescence (ECL) assay provided a wider dynamic range. Initially, an ECL 18-plex assay was used to reassess plasma samples at baseline and at 2, 4, and 6 hours after dose in the last cohort of the 16-dose study (seven received 150 μg of Nexvax2 and seven received placebo). Plasma IL-2, IL-8, MCP-1, IL-10, IFN-γ, MIP-1β, IP-10, eotaxin, and TNF-𝛼 showed significant elevations in patients receiving gluten peptides compared to placebo (Fig. 2, A and B, and table S2), but IL-1β, IL-4, IL-6, IL-12p70, IL-13, and eotaxin-3 were no different (Mann-Whitney *U* test). For IL-2, the median baseline concentration of 0.2 pg/ml [interquartile range (IQR), 0.1 to 0.2 pg/ml] was substantially below that for the magnetic bead assay (4.8: 3.2 to 15 pg/ml), but median peak concentrations at 4 hours after gluten peptides measured by each assay were similar (ECL, 23; IQR, 3.0 to 52 pg/ml; magnetic bead, 20, 7.5 to 58 pg/ml; *n* = 7). Consequently, the median fold change in IL-2 at 4 hours compared to baseline was substantially higher with the ECL assay (127; IQR, 35 to 252) compared to the magnetic bead assay (2.0; IQR, 1.0 to 11). Assessment of baseline concentrations of IL-8 was less affected, which resulted in the relative increase in IL-2 being at least 10 times greater than IL-8. In addition, the ECL assay demonstrated induction of IFN-γ at 6 hours in patients receiving gluten peptides (Fig. 2C), median plasma concentration of IFN-γ at baseline was 7.1 pg/ml (IQR, 3.7 to 11 pg/ml) compared to 26 pg/ml (IQR, 9.6 to 46) at 6 hours (median fold change, 3.2; IQR, 2 to 4.6; *P* = 0.0194, Mann-Whitney *U* test) (table S2). None of the other cytokines assessed by ECL assay showed substantial alteration in fold change from baseline when compared with magnetic bead assay. Collectively, these findings show that IL-2 is the cytokine that increases most relative to baseline after CeD patients are administered gluten peptides.

### Magnitude, consistency, and coordination of cytokine changes after gluten peptides

To better understand the magnitude, consistency, and relationship between changes in key cytokines (IL-2, IL-8, and IL-10) from baseline to 4 hours after the first dose of gluten peptides, we reevaluated plasma samples from all 54 patients who received Nexvax2 with the ECL assay. Median fold changes for IL-2, IL-8, and IL-10 were 272 (IQR, 11 to 615), 11 (IQR, 2.7 to 31), and 1.2 (IQR, 1.0 to 3.4), respectively (Table 1). A responder analysis was undertaken by establishing cutoffs corresponding to 3 SDs above the mean fold changes from baseline at 4 hours in the seven placebo-treated patients reassessed by ECL assay. Accordingly, 52 (96%) of 54 patients administered gluten peptides had an IL-2 response (cutoff, 1.6; *P* = 8.25 × 10^−8^, Fisher’s exact test), and 42 (78%) had an IL-8 response (cutoff, 2.2; *P* = 1.15 × 10^−4^) (Table 1). All seven reassessed placebo-treated patients and two Nexvax2-treated patients were nonresponders for both IL-2 and IL-8. In patient plasma samples collected after administering gluten peptides, concentrations of IL-2 at 4 hours measured by ECL assay were strongly correlated with IL-8 at 4 hours (ECL assay, *r* = 0.85; *P* = 6.7 × 10^−18^, Fisher’s exact test) (Fig. 2D), MCP-1 at 4 hours (magnetic bead assay, *r* = 0.75; *P* = 8.6 × 10^−12^) (Fig. 2E), and IP-10 at 6 hours (magnetic bead assay, *r* = 0.74; *P* = 1.5 × 10^−11^) (Fig. 2F). Combining these findings with earlier observations, IL-2 is both the earliest and most sensitive marker for the coordinated cytokine release that was almost universal in HLA-DQ2.5^+^ CeD patients administered gluten peptides.

**Table 1:** Plasma cytokines after intradermal Nexvax2 or gluten challenge assessed with ECL assay. Median, IQR values are shown. ND, not determined; NA, not applicable.

**#Patients (*N*)**		**Phase 1 trials of Nexvax2 i.d. in HLA-DQ2.5 CeD on GFD**					**Sham-controlled food** **challenge in HLA-DQ2.5** **CeD on GFD**		**Open-food challenge muesli bar (5.7 g) in** **CeD on GFD**		
		**1st dose** **Nexvax2** **All** **cohorts** **Any dose** **(60–300** **μg)**	**Nexvax2 150 μg 3-dose** **study**		**Nexvax2 150 μg 16-dose** **study**		**3-g gluten** **protein** **(vital** **wheat** **gluten** **flour** **slurry)**	**Matched** **gluten-** **free** **challenge** **(rice flour** **slurry)**	**All** **participants**	**HLA-** **DQ2.5^+^**	**HLA-DQ8^+^** **(−ve for** **HLA-DQ2.5)**
			**1st dose**	**3rd** **(final)** **dose**	**1st dose**	**16th** **(final)** **dose**					
		**54**	**8**	**8**	**15**	**15**	**11**	**8**	**19**	**16**	**3**
IL-2	Baseline, pg/ml	0.1 (0.1–0.2)	0.2 (0.1–0.3)	0.1 (0.1–0.3)	0.1 (0.1–0.2)	0.1 (0.1–0.2)	0.1 (0.1–0.1)	0.1 (0.1–0.2)	0.1 (0.1–0.3)	0.2 (0.1–0.3)	0.1 (0.1–0.2)
	Peak, pg/ml	33 (1.3–92)	40 (6.9–106)	0.9 (0.5–2.0)	53 (26–101)	0.2 (0.1–0.2)	1.8 (0.7–2.2)	0.2 (0.1–0.2)	1.0 (0.2–7.2)	0.9 (0.2–4.2)	8.0 (2.1–23)
	Peak, fold change	272 (12–597)	248 (34–707)	8.4 (2.3–22)	323 (131–915)	1.0 (1.0–1.3)	15 (5.3–27)	1.0 (1.0–1.0)	10 (1.8–27)	7.3 (2.0–19)	29 (8.4–246)
	Fisher’s exact, *P* value	8.25E-08	1.55E-04	0.0070	5.86E-06	1.0	0.0001	NA	0.0006	0.0013	0.0545
	Responder (%)	96	100	75	100	0	91	0	74	75	67
	Correlation with IL-8, *r* value	0.84	0.98	ND	0.92	ND	0.81	NA	0.71	0.74	0.17
	Correlation with IL-8, *P* value	1.66E-15	3.25E-05	ND	1.23E-06	ND	0.0027	NA	0.0006	0.0011	0.8902
	Correlation with IL-10, *r* value	0.63	ND	0.98	ND	0.43	0.84	NA	0.96	0.99	0.85
	Correlation with IL-10, *P* value	5.80E-07	ND	1.30E-05	ND	0.1120	0.0013	NA	6.26E-11	2.64E-14	0.3532
IL-8	Baseline, pg/ml	4.0 (3.1–4.9)	3.4 (2.9–5.0)	ND	3.2 (2.5–3.8)	ND	8.5 (7.2–12)	5.8 (5.2–8.6)	4.8 (3.5–5.5)	4.9 (3.8–5.8)	3.4 (2.1–4.6)
	Peak, pg/ml	43 (12–112)	34 (27–76)	ND	68 (35–168)	ND	34 (12–46)	6.5 (5.4–11)	7.0 (5.0–18)	7.0 (5.2–17)	15 (5.7–28)
	Peak, fold change	11 (2.7–31)	12 (4.7–24)	ND	13 (11–38)	ND	2.4 (1.3–4.7)	1.1 (1.0–1.2)	1.5 (1.1–4.2)	1.3 (1.1–3.3)	4.5 (2.2–6.1)
	Fisher’s exact, *P* value	1.15E-04	0.0014	ND	2.11E-04	ND	0.0128	NA	0.0258	0.0538	0.0545
	Responder (%)	78	88	ND	87	ND	64	0	47	44	67
IL-10	Baseline, pg/ml	6.1 (3.2–13)	ND	0.3 (0.2–1.1)	ND	0.2 (0.1–0.2)	0.2 (0.2–0.6)	0.3 (0.2–0.7)	0.3 (0.2–0.3)	0.3 (0.2–0.4)	0.3 (0.2–0.3)
	Peak, pg/ml	15 (6.4–41)	ND	2.3 (0.8–8.3)	ND	0.2 (0.2–0.3)	0.6 (0.4–0.7)	0.3 (0.2–0.7)	0.3 (0.3–0.7)	0.3 (0.3–0.9)	0.4 (0.2–0.4)
	Peak, fold change	1.2 (1.0–3.4)	ND	7.7 (1.9–28)	ND	1.1 (1.0–1.3)	1.5 (1.1–1.9)	1.0 (1.0–1.0)	1.2 (1.0–1.6)	1.2 (1.0–2.3)	1.2 (1.2–1.3)
	Fisher’s exact, *P* value	0.0844	ND	0.0014	ND	0.5227	0.0445	NA	0.2855	0.2622	1.0
	Responder (%)	39	ND	88	ND	20	45	0	21	25	0

### Genetic, clinical, and demographic associations with cytokine release after gluten peptides

The HLA-DQ2.5 gene dose effect in CeD is directly related to the magnitude of responses by gluten-specific T cell clones (TCCs) stimulated by cognate peptide in vitro (*18*). Analysis of plasma IL-2 and IL-8 concentrations and fold changes at 4 hours measured by ECL assay indicated a trend toward higher levels of both cytokines after receiving gluten peptides in patients who were homozygous for both HLA-DQA1*05 and HLA-DQB1*02 (IL-2: median, 49 pg/ml versus 21 pg/ml; *P* = 0.068, Mann-Whitney *U* test; IL-8: 59 pg/ml versus 37 pg/ml; *P* = 0.071) (fig. S2A).

Gluten challenge over 3 days increases peripheral blood frequencies of gluten-specific CD4^+^ T cells (*2*). Participants in ascending dose cohorts (*n* = 44), who completed a 3-day gluten food challenge at the start of the screening period, had 4-hour plasma IL-2 and IL-8 concentrations that were borderline or significantly higher than participants in the last cohorts (*n* = 10), who did not have a screening gluten challenge (IL-2: median, 35 pg/ml versus 4 pg/ml; *P* = 0.073, Mann-Whitney *U* test; IL-8: 48 pg/ml versus 11 pg/ml; *P* = 0.015) (fig. S2B). No correlation was found between the magnitude of IL-2 or IL-8 response and age, gender, physical characteristics, or time since CeD diagnosis.

### Gastrointestinal symptoms and plasma cytokines after first gluten peptide injection

Severity or occurrence of organ-specific symptoms may be a clinical proxy for cytokine levels in the source organ and in blood. Vomiting was the most common adverse event, and nausea was the most prominent gastrointestinal symptom on the first day of dosing in phase 1 clinical trials of Nexvax2 (*6*). Vomiting affected 20 of 54 (37%) patients who received gluten peptides compared to none of 28 who received placebo (*P* = 7.2 × 10^−5^, Fisher’s exact test). As measured by magnetic bead assay, cytokine elevations were exaggerated in patients receiving gluten peptides who vomited (Fig. 2G). IL-2 and IL-8 were the only cytokines to rise in plasma before the earliest onset of vomiting, which was at 2 hours and 15 min after dose (Fig. 2H). Higher nausea scores and the presence of vomiting on the first day were significantly associated with higher ECL assessments of IL-2 (Fig. 2I) and IL-8 plasma concentrations at 4 hours (fig. S2C) and with magnetic bead assay assessments of MCP-1 at 4 hours (fig. S2D) and IP-10 at 6 hours (fig. S2E). Notably, vomiting was significantly more common (19 of 37 versus 1 of 17; *P* = 0.002, Fisher’s exact test) (Fig. 2J), and self-reported daily nausea scores were higher when the IL-2 plasma concentration at 4 hours after dose exceeded 10 pg/ml.

### Gastrointestinal symptoms and plasma cytokines after multiple gluten peptide injections

If cytokine release and symptoms following the first administration of gluten peptides depend on activation of CD4^+^ T cells, then both could be expected to moderate with repeat administrations of gluten peptides because persistent antigenic exposure reduces responsiveness of CD4^+^ T cells (*19*).

In the three-dose study, digestive symptoms were triggered by the second and third doses of gluten peptides given at weekly intervals, albeit less than the first dose (*6*). Plasma collected from patients after their third 150-μg dose was assessed using the 38-plex magnetic bead assay. With two notable exceptions, cytokine elevations after the third dose of gluten peptides were markedly attenuated compared to the first dose (Fig. 1B). IL-10 induction was preserved and was similar for the third and first doses. In contrast, IL-2 elevations after the third dose could only be detected using the ECL assay and were found to be 8.4-fold increased at 4 hours compared to baseline, but this was approximately 40 times less than the 248-fold increase after the first dose (Table 1). The only patient who vomited after the third weekly dose of 150 μg of Nexvax2 had the third highest IL-2 plasma concentration at 4 hours and was among four who had plasma concentrations of IL-2 between 1.0 and 15 pg/ml (Fig. 2K).

In the 16-dose study when gluten peptides were injected at intervals of 3 or 4 days over 8 weeks, vomiting was absent, and gastrointestinal symptoms and adverse events were seldom experienced after the fourth and later doses (*6*). The plasma cytokine profile in patients after the 16th dose of gluten peptides was no different from patients receiving placebo (Fig. 1C), which was confirmed for IL-2 by finding that concentrations were uniformly less than 0.5 pg/ml when plasma samples were reassessed by ECL assay (Fig. 2L and Table 1). Together, these findings indicate attenuation of both cytokine release and symptoms with repeat doses of gluten peptides, which is consistent with induction of CD4^+^ T cell unresponsiveness to gluten peptides.

### Evaluation of plasma and serum cytokines after ingestion of gluten

Gluten is a complex mixture of poorly soluble proteins that, after partial digestion and absorption in the proximal digestive tract and TG2-mediated deamidation in the intestinal mucosa, is likely to generate a diverse range of peptides of variable length including some that are covalently linked to TG2 itself (*2*). Hence, peptides resulting from gluten ingestion are likely to have more prolonged and diverse effects than the short, soluble deamidated gluten peptides in Nexvax2. However, it might be expected that the CD4^+^ T cells activated by gluten peptides would also be activated after ingestion of gluten. A double-blind, sham-controlled gluten food challenge was undertaken in 19 HLA-DQ2.5^+^ CeD patients on GFD. Patient characteristics are shown in table S3. The active food challenge contained 3 g of gluten protein.

**Fig. 3:**
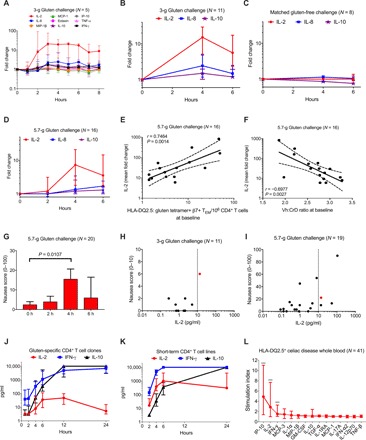
Validation of IL-2 activation in gluten-induced recall responses. (**A** to **C**) Fold-change responses assessed in CeD patients in a randomized double-blind 3-g gluten food challenge study with matched controls. Median and IQRs are shown. (**D**) IL-2 response to gluten challenge from stored plasma samples collected in a previously reported open-label 5.7-g study. Median and IQRs of response in HLA-DQ2.5^+^ patients are shown. (**E** and **F**) Pearson’s correlation analysis of IL-2 activation with frequency of circulating gluten-specific CD4^+^ T cells in HLA-DQ2.5^+^ CeD patients (E) and villous height–to–crypt depth (Vh:CrD) ratio (F) at baseline before oral gluten challenge. (**G**) Self-reported nausea scores collected every 2 hours after gluten challenge. Median with IQRs is shown. *P* value was estimated by paired Wilcoxon signed-rank test. (**H** and **I**) Dose-response analysis of IL-2 concentration with patient-reported nausea scores and occurrence of vomiting in double-blind 3-g gluten food challenge study (H) and previously reported gluten challenge study (I). Red dot indicates that patient vomited after gluten challenge. (**J**) Cytokine response of CD4^+^ T cell clones, specific for HLA-DQ2.5–restricted epitopes in Nexvax2 and derived from peripheral blood or intestinal biopsies of CeD patients, after incubation with anti-CD3 and anti-CD28 antibody in the absence of other cell types for 24 hours. (**K**) Cytokine response of short-term CD4^+^ T cell lines, derived from peripheral blood or intestinal biopsies of CeD patients, after incubation with anti-CD3 and/or anti-CD28 antibody in the absence of other cell types for 24 hours. (**L**) Cytokine response in fresh whole blood, drawn from HLA-DQ2.5^+^ CeD patients, incubated with Nexvax2 peptides for 24 hours. Stimulation index estimated as fold change relative to incubation with media only. Median value and IQR are shown. Significance of cytokine induction estimated by paired Wilcoxon signed-rank test between Nexvax2 and Nil incubations. Asterisks denote cytokines with significant differences (***P < 0.0001).

Among the 18 cytokines tested by ECL assay in the first six patients, IL-2, IL-8, and IL-10 showed the greatest fold change from baseline beginning from 2 hours and were at maximal levels typically between 4 and 6 hours after gluten (Fig. 3A). Serum and plasma measurements were correlated for each of these three cytokines (fig. S3), which enabled assessment of serum alone and testing only by ECL assay for IL-2, IL-8, and IL-10 for subsequent patients. Together, in the group of 11 patients who consumed gluten, IL-2, IL-8, and IL-10 were significantly elevated at 4 and/or 6 hours compared to 8 participants after matched gluten-free challenge (Fig. 3, B and C). According to responder analysis, on the basis of a threshold level established in sham-challenged participants as it previously was for gluten peptide administration, 10 (91%) of the patients who consumed gluten were IL-2 responders, compared to 7 (64%) for IL-8 and 5 (45%) for IL-10, whereas none of the patients who consumed the gluten-free food challenge were responders to any cytokine (Table 1). Median fold change at 4 hours after ingestion of gluten was 15 for IL-2 (IQR, 5.3 to 27) (Table 1), which was 6 times more than for IL-8, and 10 times greater than IL-10. Plasma IL-2 concentrations at 4 hours correlated with elevations in IL-8 (Pearson *r* = 0.81, *P* = 0.0027) and IL-10 (*r* = 0.84, *P* = 0.0013). Together, the serum cytokine profile following gluten ingestion is less prominent but qualitatively similar and over a corresponding time course to that after injecting gluten peptides, which is consistent with activated CD4^+^ T cells being the driver of cytokine release in both scenarios.

### Gluten-specific CD4^+^ T cell frequency and plasma cytokines after ingestion of gluten

Frozen plasma from a previously reported study provided the opportunity to explore whether cytokine release after gluten food challenge in CeD patients on GFD correlated with baseline duodenal histology and the frequency of gluten-specific CD4^+^ T cells in blood (*5*). Plasma from blood collected on the first day of the study at baseline and at 2, 4, and 6 hours after participants consumed a muesli bar containing 5.7 g of gluten were analyzed for IL-2, IL-8, and IL-10 by ECL assay. Statistically significant elevations in IL-2, IL-8, and IL-10 were similar in magnitude and over a time course comparable to the 3-g gluten food challenge (Fig. 3D and Table 1). Cytokine elevations in three HLA-DQ8^+^ CeD patients, who were negative for HLA-DQ2.5, were similar to 16 HLA-DQ2.5^+^ CeD patients (Table 1). At 4 hours, IL-2 levels were correlated with IL-8 (Pearson *r* = 0.71, *P* = 6.34 × 10^−4^) and IL-10 (*r* = 0.96, *P* = 6.26 × 10^−11^). In HLA-DQ2.5^+^ patients, the gluten-specific T cell response, as evaluated by flow cytometry after tetramer staining, was positively correlated with average plasma concentrations of IL-2 at 2, 4, and 6 hours after gluten challenge (Pearson *r* = 0.75, *P* = 0.0014) (Fig. 3E) and showed similar trends with IL-8 (*r* = 0.77, *P* = 8.444 × 10^−4^) and IL-10 (*r* = 0.53, *P* = 0.0439). In addition, duodenal villous height-to-crypt depth ratio was negatively correlated with average plasma IL-2 concentrations after gluten challenge (Pearson *r* = −0.69, *P* = 0.0027) (Fig. 3F) and showed similar trends with IL-8 (*r* = −0.59, *P* = 0.0162) and IL-10 (*r* = −0.62, *P* = 0.01).

### Linking gastrointestinal symptoms to plasma cytokines after ingestion of gluten

After 3- and 5.7-g gluten food challenges in two separate studies, symptoms were milder than in phase 1 studies of Nexvax2 with nausea being the only individual symptom in either food challenge study that increased significantly (at 4 hours) from baseline and only in the patients who consumed 5.7 g of gluten (Fig. 3G). However, IL-2 plasma concentrations trended upward with greater nausea at 4 hours after challenge in both studies (Fig. 3, H and I). One patient in each study vomited, in both cases, vomiting was during the third hour after gluten, and both affected patients were among the five with IL-2 plasma levels above 10 pg/ml at 4 hours (Fig. 3, H and I). Together, these findings demonstrated that vomiting and more severe nausea after ingesting gluten were associated with greater cytokine release after ingesting gluten.

### In vitro cytokine secretion by gluten-specific CD4^+^ TCCs

IFN-γ has been considered the dominant cytokine secreted by gluten-specific CD4^+^ T cells (*2*). Since the kinetics of IL-2 secretion by antigen-stimulated memory T cells is known to be rapid and transient (*20*), we speculated that IL-2 secretion by gluten-specific CD4^+^ TCCs might have been overlooked because of long incubation periods (*21*). A time course study of in vitro–activated gluten-specific CD4^+^ TCCs incubated in the absence of other cells was undertaken.

Intestinal and blood-derived gluten-specific CD4^+^ TCCs were raised in the absence of gluten antigen and underwent three to seven rounds of stimulation and expansion at intervals of 7 to 10 days. On day 9 of the last stimulation, isolated gluten-specific CD4^+^ TCCs were stimulated with monoclonal antibodies specific for CD3 and CD28 or with gluten peptide–HLA-DQ2.5 complexes to provide potent stimulation via the T cell receptor complex. Media collected after 1 to 24 hours were analyzed using the 38-plex magnetic bead assay. IL-2 secretion was present in 9 of 11 TCCs. IL-2 concentrations in media were maximal at 6 to 12 hours, but IL-2 concentrations were reduced to near the lower limit of detection by 24 hours in most TCCs, likely due to consumption of this autocrine T cell growth factor by the proliferating cells (Fig. 3J). In contrast, all TCCs secreted both IFN-γ and IL-10 with their concentrations steadily increasing over 24 hours and for many clones reaching levels above the limits of quantitation (fig. S5). Overall, concentrations of IFN-γ, IL-10, IL-4, granulocyte-macrophage colony-stimulating factor (GM-CSF), MIP-1α, MIP-1β, TNF-α, and often IL-5 and IL-8 were near to or above the upper level of quantitation in incubation media from most gluten-specific CD4^+^ TCCs after stimulation for 6 hours (fig. S6).

To address the possibility that cytokine secretion of activated CD4^+^ T cells may be altered by the absence of professional antigen-presenting cells, we incubated six separate gluten-specific CD4^+^ TCCs with cognate gluten peptide and DCs derived from a healthy HLA-DQ2.5^+^ donor. Peptide presentation by DCs did not change the set of cytokines that could be attributed to activated gluten-specific CD4^+^ T cells but did highlight that unstimulated DCs alone were capable of secreting IL-2 and high levels of IP-10 and MDC/CCL22 without addition of peptide (fig. S6). Together, these experiments confirmed that activated gluten-specific CD4^+^ TCCs secrete IL-2, but it is transient and modest compared to IFN-γ.

### Cytokine secretion by short-term CD4^+^ T cells and fresh blood from CeD donors

In vitro responses of CD4^+^ TCCs may not accurately reflect the function of CD4^+^ T cells in vivo (*22*). We speculated that IL-2 secretion relative to other cytokines might be more prominent in CD4^+^ T cell lines cultured for relatively short periods.

Intestinal and peripheral blood–derived CD4^+^ T cells were isolated from CeD donors and, over 29 days, underwent a single round of expansion. Culture media were collected following stimulation of T cell lines for up to 24 hours with anti-CD3 and anti-CD28. At 6 hours, the range of cytokines produced by these short-term CD4^+^ T cell lines was similar to gluten-specific CD4^+^ TCCs except that IL-2 production was more prominent (fig. S6). Median concentrations of IL-2 and IL-10 were 29% (IQR, 13 to 45%; *n* = 4) and 3% (IQR, 1.2 to 5.2%; *n* = 4) of the median concentration of IFN-γ (Fig. 3K and fig. S6). This was compared with median net concentrations of IL-2 and IL-10 that were 4% (IQR, 0.4 to 15%; *n* = 11) and 78% (IQR, 9 to 265%), respectively, of the median net IFN-γ concentration after gluten-specific TCCs were incubated for 6 hours with anti-CD3 and anti-CD28 (fig. S5).

To more directly test whether unmanipulated gluten-specific CD4^+^ T cells secrete IL-2, we used the 38-plex magnetic bead assay to assess cytokine profiles in stored plasma from heparinized whole blood (1 ml) incubated with Nexvax2 gluten peptides [each at 50 μg/ml in phosphate-buffered saline (PBS)] or PBS alone for 41 CeD patients before and 6 days after 3-day gluten food challenge during the screening period of phase 1 clinical trials of Nexvax2 (*6*).

For blood collected before gluten food challenge and incubated with gluten peptides, there were no cytokines showing significant elevation compared to incubation with media alone (fig. S7). By contrast, after gluten food challenge, when frequencies of memory gluten-specific CD4^+^ T cells are increased (*2*), gluten peptides stimulated significant elevations in IL-2, IFN-γ, and IP-10 (Fig. 3L and fig. S7). Median plasma concentration of IL-2 in blood incubated with gluten peptides was 22 pg/ml (IQR, 9 to 41) compared to 4.3 pg/ml (IQR, 3.2 to 9.6) for media alone (median fold change, 2.9; IQR, 1.0 to 7.7; *P* = 0.0027; paired Wilcoxon test). Median plasma concentration of IFN-γ in blood incubated with gluten peptides was 74 pg/ml (IQR, 34 to 156) compared to 27 pg/ml (IQR, 19 to 77) for media alone (median fold change, 1.5; IQR, 1.1 to 3.3; *P* = 0.0055). Median plasma concentration of IP-10 in blood incubated with gluten peptides was 4242 pg/ml (IQR, 1339 to 10,000) compared to 696 pg/ml (IQR, 545 to 1034) for media alone (median fold change, 4.9; IQR, 1.4 to 11; *P* = 0.0005). Gluten peptide–stimulated concentrations of IL-2 and IFN-γ and also IP-10 and IFN-γ were significantly correlated (IL-2 and IFN-γ, Pearson *r* = 0.90; *P* = 9.11 × 10^−16^; IFN-γ and IP-10, *r* = 0.72; *P* = 1.15 × 10^−07^). Median net increase in IL-2 concentration relative to IFN-γ was 36% (IQR, 9 to 80%; *n* = 41) in plasma from fresh blood incubations with Nexvax2 gluten peptides compared to media alone.

Together, IL-2 secretion is a prominent feature in the cytokine profile when fresh blood enriched for gluten-specific CD4^+^ T cells is stimulated with gluten peptides, which is in keeping with IL-2 secretion by activated short-term CD4^+^ T cell lines.

### In vitro cytokine secretion by freshly isolated B cells

Last, the cytokine profiles of freshly isolated B cells were investigated because gluten- and TG2-specific B cells are implicated in CeD, and B cells and plasma cells are the most abundant cell type presenting the immunodominant gluten peptide DQ2.5-glia-α1a in the inflamed intestinal tissues of CeD patients (*23*). B cells were isolated from fresh peripheral blood mononuclear cells (PBMCs) of healthy individuals (*n* = 3) by negative selection. After B cell enrichment, 99% of cells were CD19^+^. These B cell preparations were stimulated with IL-4, sCD40L, and anti-human immunoglobulin for 24 hours. Incubation media were analyzed using the 38-plex magnetic bead assay. MDC/CCL20 was consistently above the upper levels of quantitation (10,000 pg/ml), and concentrations of IL-6 were between 500 and 1000 pg/ml, but IL-2 was below levels of detection (3.2 pg/ml), and concentrations of other cytokines were less than 150 pg/ml (fig. S6). Together, these and previous in vitro findings strongly suggest that activated B cells and DCs secreting MDC/CCL20 did not make a substantial contribution to the cytokines elevated in plasma after gluten ingestion or administration of gluten peptides.

## DISCUSSION

These studies addressed the immunological basis for early digestive symptoms experienced by CeD patients after intradermal injection of gluten peptides and also after physiologic gluten ingestion. Rapid, coordinated elevation of circulating cytokines including IL-2 confirmed in vivo activation of CD4^+^ T cells in CeD patients after injecting short antigenic gluten peptides or ingestion of gluten that was closely associated with the onset and severity of acute digestive symptoms. These findings were notable because the cytokine signature after injecting gluten peptides that can potentially bind directly to HLA-DQ2.5 on professional antigen-presenting cells mimicked that caused by ingesting gluten, although peptides derived from digestion of gluten in the gut need to be absorbed, require partial deamidation for immune presentation, are more diverse, and have the additional properties of efficiently activating humoral immunity and causing chronic intestinal injury.

The gluten peptides we studied correspond to partially deamidated 15– or 16–amino acid fragments of wheat α- and ω-gliadin proteins and barley B-hordein protein that harbor altogether six HLA-DQ2.5–restricted epitopes known to activate most of the gut- and blood-derived gluten-specific CD4^+^ T cells in HLA-DQ2.5^+^ CeD patients (*7*, *8*). This peptide mixture is being investigated as a potential immunotherapy (Nexvax2) for CeD by exploiting the immunomodulatory properties of immunodominant CD4^+^ T cell epitopes (*24*). We have previously reported in detail clinical aspects of two parallel fixed, intradermal, ascending dose phase 1 studies of Nexvax2 (*6*). In contrast to our limited understanding of the immune basis for T cell–mediated autoimmune disease, these clinical studies provided the opportunity to test the clinical effects of selectively engaging and activating pathogenic CD4^+^ T cells in patients. For CeD, this aspect was particularly significant because previously the causative antigen, gluten, could only be administered to the gut, which made it impossible to distinguish direct topical immunotoxic effects from those mediated by activated gluten-specific CD4^+^ T cells. Furthermore, our understanding of gluten-specific CD4^+^ T cell function has been limited to studies reliant on in vitro/ex vivo assays or nonhuman models, reductionist approaches that have been increasingly scrutinized (*22*).

The systemic cytokine release observed provides definitive evidence of rapid immune activation within 2 hours after administering gluten peptides in almost all HLA-DQ2.5^+^ CeD patients. Qualitative and quantitative assessments of cytokines elevated after injecting gluten peptides or gluten food challenge were complicated by individual cytokines having different temporal profiles and by low baseline concentrations of key cytokines such as IL-2. Ultimately, three different multiplex assay platforms screening for up to 92 cytokines were used to show statistically significant, coordinated elevations in plasma IL-2, IL-8, MCP-1, IL-10, MIP-1β, IP-10, and eotaxin after injection of gluten peptides. High-sensitivity assays for key analytes at select time points showed that the earliest cytokines to rise and reach peak levels in plasma included IL-2, which was also the cytokine to show most prominent elevations. These findings are in keeping with the kinetics of CD4^+^ T cell activation and cytokine release when short, soluble antigenic peptide is administered in murine T cell transfer models (*22*).

Although gluten peptides were not delivered to the gut, gastrointestinal symptoms mimicking those after gluten exposure were observed, and their onset occurred when plasma cytokine levels were rising. More severe nausea with or without vomiting was associated with higher peak plasma cytokine levels. The link between immune activation and symptoms was further strengthened by showing that postdose symptoms and cytokine release were both lessened after three weekly doses and absent after 16 twice-weekly injections of gluten peptides. These findings are consistent with the difference in severity of symptoms after gluten ingestion compared to gluten peptide injection being related to potency of the antigen challenge and T cell activation measured by circulating IL-2 concentration at 4 hours.

Prompted by these observations, we investigated whether systemic cytokine release occurred in CeD patients after “one-off” food challenges containing either 3 or 5.7 g of gluten, amounts equivalent to about one quarter or one half of the average daily intake of gluten in the United States (*25*). In contrast to previously reported findings using a less sensitive assay format (*5*), cytokine release dominated by elevation of IL-2 could be detected from as early as 2 hours after gluten ingestion. Cytokines showing statistically significant elevations among the set of 18 tested were limited to IL-2, IL-8, and IL-10. The prominence of IL-2 followed by IL-8, their temporal profiles, and tight correlation closely resembled their release after injecting gluten peptides. However, peak cytokine elevations were substantially less after gluten ingestion than after injection of gluten peptides. In keeping with gluten-specific CD4^+^ T cells being responsible for cytokine release after gluten exposure, plasma IL-2 elevations were correlated with peripheral blood frequencies of these cells. We found that gluten challenge with 5.7 g of gluten significantly increased nausea, which peaked at 4 hours. More severe nausea and vomiting were associated with higher plasma concentrations of IL-2.

Implicating a cellular source for IL-2 elevations in plasma after ingesting gluten or injecting gluten peptides is a key mechanistic question. Activated T cells are the primary source of IL-2, but DCs can also secrete IL-2 following ligation of specific pathogen recognition receptors; mast cells also secrete IL-2 following exposure to IL-33 or IL-9 (*26*, *27*). CeD is hallmarked by CD4^+^ T cells that recognize deamidated gluten epitopes (*2*), and most of the gluten-specific CD4^+^ T cells in HLA-DQ2.5^+^ CeD patients are reactive with the epitopes represented in Nexvax2 (*7*). The IL-2 elevation being early and prominent in the cytokine signature after injecting Nexvax2 gluten peptides in CeD patients is in keeping with the known kinetics of IL-2 secretion by memory CD4^+^ T cells in vivo (*20*). CD4^+^ T cells specific for gluten are enriched in intestinal tissue and may also be present in gut-associated secondary lymphoid organs, but in blood from HLA-DQ2.5^+^ CeD patients on GFD memory, CD4^+^ T cells specific for epitopes in Nexvax2 have a median frequency estimated to be only 4 per million CD4^+^ T cells (*5*). This frequency is much lower than in T cell transfer models in mice and is likely to preclude direct assessment of IL-2 secretion by gluten-specific CD4^+^ T cells collected from patients after injecting gluten peptides or ingesting gluten.

The unexpectedly modest changes in blood levels of IFN-γ, a cytokine historically considered as the archetypical marker for activated gluten-specific CD4^+^ T cells (*2*), after ingesting gluten or gluten peptide, administration may have been contributed to by the relatively high baseline concentrations of this cytokine compared to IL-2. By contrast, the in vitro experiments with activated gluten-specific intestinal and blood-derived CD4^+^ TCCs demonstrated only transient secretion of IL-2 that was modest compared to the sustained secretion of IFN-γ and several other cytokines. This likely relates to the fact that IL-2 is an autocrine growth factor for T cells, which, unlike many other cytokines, is consumed by proliferating TCCs in dense cultures in vitro (*28*). Further, reliance on long-term TCCs to predict how memory T cells participate in the in vivo immune response has been questioned (*22*). Therefore, we used fresh blood from CeD patients collected 6 days after the gluten food challenge to provide a source of fresh, unmanipulated gluten-specific CD4^+^ T cells likely to mimic in vivo cytokine release more closely than long-term TCCs (*2*). IL-2, IFN-γ, and IP-10, a monocyte-derived chemokine whose expression was closely correlated to elevations in IFN-γ, were selectively elevated following 24-hour incubation of Nexvax2 gluten peptides with fresh blood collected from HLA-DQ2.5^+^ CeD patients after, but not before, gluten food challenge. The known properties of IL-2 and IFN-γ, and their closely correlated secretion in fresh blood collected from CeD donors, are consistent with their source being gluten-specific CD4^+^ T cells. That cytokine secretion by unmanipulated gluten-specific CD4^+^ T cells in fresh blood from CeD patients that is biased to IL-2 and/or IFN-γ is in keeping with fresh blood recall CD4^+^ T cell responses to viral, fungal, and bacterial recall antigens (*29*). Our findings highlight that cytokine secretion by gluten-specific CD4^+^ T cells in patients is unlikely to be accurately reflected by in vitro stimulation of TCCs. Furthermore, these findings add support to the conclusion that gluten-specific CD4^+^ T cells are the source of elevated blood IL-2 and IFN-γ levels following gluten ingestion or administration of gluten peptides.

Whether cytokines elevated in blood after injecting gluten peptides or ingesting gluten have any direct extra-intestinal effects is unclear. Fatigue, headache, and “brain fog” are the commonly reported extra-intestinal symptoms in CeD patients (*3*). However, symptoms being focused on the upper gastrointestinal tract suggest that cytokines increased in blood have clinical and immunological effects that selectively affect the tissue from which they originate. The small intestinal mucosa in CeD is infiltrated with gluten-specific CD4^+^ T cells and B cells and plasma cells specific for gluten and TG2, which are thought to facilitate highly efficient presentation of gluten peptides to gluten-specific CD4^+^ T cells (*23*). The prominence of IL-2 and the absence of MDC/CCL22, a chemokine derived from activated professional antigen-presenting cells, argue for activated CD4^+^ T cells rather than B cells in the adaptive immune response to gluten being responsible for initiating cytokine release after injecting gluten peptides or ingesting gluten.

Nausea and vomiting are common adverse effects of high-dose recombinant IL-2 given as a bolus injection (*30*). After subcutaneous administration of high-dose IL-2, peak 4-hour serum levels are approximately 4 ng/ml (*31*). This is 400 times higher than the threshold IL-2 level (10 pg/ml) at 4 hours after intradermal Nexvax2 or gluten ingestion above, which vomiting and more severe nausea were common. However, since the duodenum is unusually rich in serotonin-containing enterochromaffin cells, and receptors for bioactive amines in the duodenal mucosa play a key role in controlling nausea and vomiting (*32*), high local tissue concentrations of IL-2 or downstream mediators after reactivation of gluten immunity in the duodenal mucosa may be sufficient to trigger these symptoms without prominent systemic effects. Future studies should test whether cytokine concentrations are substantially higher in gut mucosal tissue than in blood after gluten challenge or injection of gluten peptides and determine whether alterations in local cytokine levels are matched by immune and inflammatory cell infiltration.

Our findings reveal that gluten-specific CD4^+^ T cells are rapidly activated by gluten and position these T cells as the driver of acute gut-specific symptoms in CeD. The study marks the first occasion when peptides containing immunodominant epitopes for CD4^+^ T cells in an organ-specific immune disease have been shown to stimulate rapid systemic cytokine release in patients. Postdose digestive symptoms and cytokine release do not occur when Nexvax2 doses as high as 900 μg are preceded by dose escalation from a low starting dose (*10*). The kinetics of the cytokine and symptom response and the absence of local skin reactions to intradermal gluten peptides that are rapidly cleared into the systemic compartment contrasts to conventional delayed hypersensitivity reactions, for example, to intradermal tuberculin (*11*). For understanding the pathophysiology of CeD, these findings are of major significance because the previously unappreciated importance of IL-2 shifts focus to the gluten-specific CD4^+^ T cell being responsible for early immune events and clinical symptoms after gluten exposure.

## MATERIALS AND METHODS

### Phase 1 Nexvax2 clinical trials

The clinical procedures, outcomes, and associated laboratory methods for the 3- and 16-dose randomized, double-blind, placebo-controlled, ascending dose phase 1 studies have been described in detail elsewhere (*6*). All participants provided written informed consent. Ethics committees gave approval for the three-dose study [Liberty Institutional Review Board (IRB) tracking number 12.07.0012, The University of Oklahoma Institutional Review Board for the Protection of Human Subjects IRB number 1370, Bellberry Human Research Ethics Committee application number 2013-10-553, and Southern Health and Disability Ethics Committee 13/STH/168] and for the 16-dose study (The Alfred Hospital Ethics Committee approval number 118/12, Bellberry Human Research Ethics Committee application number 2012-04-735-AA, Southern Health and Disability Ethics Committees ethics ref. NTY/12/06/049/AM05).

Stored plasma samples and clinical data were analyzed after the trials were completed and unblinding of data. Briefly, adult HLA-DQ2.5^+^ CeD patients on GFD were recruited at 12 sites in Australia, New Zealand, and the United States. During the screening period, patients evaluated for ascending dose cohorts had a 3-day gluten food challenge. Blood collected before and 6 days after commencing the gluten challenge were assessed in a whole-blood IFN-γ release assay, described in detail elsewhere (*6*). The last cohort in each study had a gastroscopy instead of a food challenge and was excluded if villous atrophy was present. Fixed doses of investigational product were administered by intradermal injection. Dose levels assessed were 60, 90, and 150 μg in the three-dose study and 150 and 300 μg in the 16-dose study. The matched placebo was 0.1 ml of sterile 0.9% sodium chloride. Timing and severity of adverse events were recorded. Gastrointestinal symptoms of pain, hunger pains, nausea, rumbling, bloating, and diarrhea were assessed daily by patients using a seven-point graded Likert scale, where one represented the most positive option, and seven represented the most negative one in the format of the Gastrointestinal Symptom Rating Scale. Blood for plasma cytokine assessment was collected into K2 EDTA Vacutainer tubes (Becton Dickinson, Franklin Lakes, NJ), which were centrifuged within 10 min of collection at 1100 to 1300 RCF (relative centrifugal force) for 10 min, and then, plasma was stored at or below −60°C. Cytokines were assessed in plasma from blood collected within 30 min before (baseline) and at 10, 20, 30, and 45 min and 1, 1.5, 2, 4, and 6 hours after dosing on the first day. Plasma over the same time course was also assessed in patients after their last dose for those in cohorts receiving the maximum tolerated dose of Nexvax2 (150 μg).

### Food challenge studies with gluten

Participants provided written informed consent. The Human Research Ethics Committee Melbourne Health (2003.009) and The Walter and Eliza Hall Institute Human Research Ethics Committee (03/04) approved the study. Male and female patients aged 18 to 70 years with biopsy-confirmed CeD following GFD for at least 8 weeks were eligible. Patients were excluded if their score was over 12 in the Celiac Dietary Adherence Test (*33*), serum TG2 immunoglobulin A (IgA) and deamidated gliadin peptide IgG were both elevated, immunomodulators or immunosuppressive medications were used during the previous 2 months, oral or parenteral corticosteroids were used within the previous 6 weeks, or females were pregnant, lactating, or breastfeeding. A double-blind food challenge consisting of either 5 g of vital wheat gluten flour (Bob’s Red Mill Natural Foods Inc., Milwaukie, OR), which was estimated to contain 3 g of gluten protein according to the Osborne equation, or gluten-free fine-ground white rice flour (“McKenzie’s Rice Flour,” Ward McKenzie’s Pty Ltd., Australia) was added to one serve of (7 g) gluten-free “Vitafresh Low Calorie Lime” or “Vitafresh Low Calorie Sweet Navel Orange” (Hansells Food Group, New Zealand) mixed together in 100 ml of water consumed over 2 min. Adverse events were recorded hourly and graded according to “The Common Terminology Criteria for Adverse Events (CTCAE) version 4.0” (U.S. Food and Drugs Administration Guidance for Industry: Toxicity Grading Scale). Each hour, a modified version of the CeD patient reported outcome questionnaire (CeD PRO) was completed by participants to grade their worst experience for 11 symptoms over the previous 1-hour graded on a whole-number scale from 0 (none) to 10 (worst possible) (*34*). Blood was collected before and hourly up to 8 hours in the first six patients, and for later patients, only at baseline and at 4 and 6 hours. Blood for plasma was collected and processed as described for phase 1 clinical studies. Blood for serum was collected into Vacutainer Plus plastic serum tubes (BD 367986). Serum was separated from blood after 2 hours at room temperature by centrifuging at 2000*g* for 20 min.

The clinical procedures, outcomes, and associated laboratory methods for a second, separate unmasked gluten food challenge have been reported in detail elsewhere (*5*). Participants were on GFD and in histological and serological remission. Quantitative histology was determined in baseline duodenal biopsies. Participants consumed one 50-g muesli bar daily that contained 7.6 g of gluten flour (5.7 g of gluten protein) and was free of fermentable oligosaccharides, disaccharides, monosaccharides, and polyols. Blood samples were collected at baseline and at 2, 4, and 6 hours after consuming the muesli bar on day 1. Plasma from these blood samples was kept frozen at −80°C and later analyzed for cytokines using a magnetic bead assay, and results have been previously reported (*5*). For the present study, the ECL assay was used to reassess IL-2, IL-8, and IL-10 in frozen plasma samples from the first study day. Symptoms were recorded by patients on a visual analog scale at baseline and then every 2 hours up to 6 hours after consuming gluten on the first study day. The frequency of effector-memory gut-homing CD4^+^ cells specific for DQ2.5:glia-α1a, DQ2.5:glia-α2, DQ2.5:glia-ω1 and DQ2.5:glia-ω2, and DQ8:glia-α1 and DQ8:glia-γ1b epitopes was assessed in blood collected at baseline according to previously described methods (*35*). These data have been previously published (*5*).

### Peripheral blood and intestinal gluten-reactive TCCs

Volunteers provided written informed consent. The Regional Committee for Medical and Health Research Ethics South East Norway (REK 2010/2720) approved the study. Intestinal TCCs were prepared from duodenal biopsies from CeD patients as previously described (*36*). To obtain peripheral blood TCCs, PBMC from CeD patients were stained with HLA-DQ2.5:gluten peptide tetramers (peptide-loaded major histocompatibility complex molecules, pMHC) labeled with phycoerythrin, sorted, and cloned by limited dilution according to previously reported methods (*37*). Six gut-derived and six blood-derived TCCs were considered to be clonal as judged by pMHC staining of >99% of cells. TCCs were seeded (1.5 million per well) into 48-well plates precoated with anti-CD3 (5 μg/ml; 300414, BioLegend, San Diego, CA) and incubated for 24 hours in 1.5 ml of RPMI 1640 supplemented with 10% heat-inactivated human serum and penicillin-streptomycin containing anti-CD28 (1 μg/ml; 302914, BioLegend). Medium (0.1 ml) was removed and centrifuged, and supernatants were frozen. In parallel, eight TCCs were also cultured for 24 hours with cognate peptide and monocyte-derived DCs derived from a HLA-DQ2.5^+^ donor not affected by CeD. DCs were prepared by incubating positively selected PBMC CD14^+^ cells (CD14 microbeads; Miltenyi Biotec Inc., San Diego, CA) with GM-CSF (1000 U/ml) and IL-4 (500 U/ml). Cell cultures were replenished on alternate days with half the medium replaced with RPMI supplemented with 10% fetal calf serum containing GM-CSF and IL-4. On day 6, lipopolysaccharide (150 ng/ml) was included with GM-CSF and IL-4 in the medium added. On day 7, mature DCs were collected, washed, counted, and resuspended in RPMI + 10% human serum (0.5 million/ml) and incubated overnight with PBS or matched cognate peptide, either 10 μM DQ2·5-glia-γ1 peptide (YQQLPQPEQPQQSFPEQERPF) or 2 μM α-gliadin-33mer peptide (LQLQPFPQPELPYPQPELPYPQPELPYPQPQPF). Peptide-pulsed DCs (0.4 million) and TCCs (1.2 million) were added to each 48 well in a total volume of 1.2 ml. Medium (0.1 ml) was removed and centrifuged, and supernatants were frozen. Six TCCs were incubated in wells coated with immobilized epitope-specific pMHC (*38*). TCCs (260,000) were incubated with plate-bound pMHC in nine replicate wells (225 μl per well). Thirty microliters of medium was removed from each of three wells and pooled, producing three pooled samples for each time point that were frozen.

### Peripheral blood and intestinal T cell lines

Volunteers provided written informed consent. The University of Chicago IRB (12623B) approved the study. PBMC were isolated by Ficoll-Paque density centrifugation. Cells were isolated from duodenal biopsies of patients with CeD on GFD via 1-hour mechanical disruption in RPMI containing 2 mM EDTA (46-034-CI, Corning) and 1.5 mM MgCl_2_ (BP214-500, Thermo Fisher Scientific) for acquisition of intraepithelial lymphocytes and subsequent 1-hour mechanical disruption in RPMI containing collagenase type IV (C5138-100, Sigma-Aldrich) (*39*) for acquisition of lamina propria lymphocytes. Cells staining positive for anti-CD3 (PE-Cy7, 300420, UCHT1, BioLegend), TCRαβ (αβ T cell receptor; BV421, 306722, IP26, BioLegend), and CD4 (PerCP/Cy5.5, 317428, OKT4, BioLegend) were purified by fluorescence-activated cell sorting (fig. S4). Five to 10,000 sorted cells were expanded with phytohemagglutinin-L (1 μg/ml; M5030, Calbiochem/EMD Millipore, Billerica, MA) and a mixture of irradiated heterologous PBMCs and Epstein Barr virus–transformed B lymphoblastoid cell lines in RPMI + 10% human serum AB (S40110, Atlanta Biologicals, Norcross, GA) and maintained with IL-2 (100 U/ml; 136, National Institutes of Health AIDS Reagent Program) (*40*). One round of cell expansion for 29 days was performed. Cells were 98.5 to 99.5% TCRαβ/CD4 double positive. For stimulation with anti-CD3 (555329, BD Bioscience, Franklin Lakes, NJ) and anti-CD28 (555726, BD Bioscience), 96-well flat-bottom plates (07-200-656, Thermo Fisher Scientific) were coated overnight (anti-CD3, 1.5 μg/ml; anti-CD28, 1 μg/ml) at 4°C. Cells were then plated 200 μl per well in RPMI + 10% human serum AB (S40110, Atlanta Biologicals, Norcross, GA) at a concentration of 1 × 10^6^ cells/ml counted using a hemocytometer and incubated 24 hours at 37°C. Supernatants were harvested and frozen at −80°C until evaluation.

### Whole-blood cytokine release

Whole-blood cytokine release has been described in detail elsewhere (*6*). Briefly, 1 ml of blood was collected into Nil Control Tubes (QuantiFERON-TB Gold In-Tube, QIAGEN, Hilden, Germany) that had 0.1 ml of PBS alone or with the mixture of three gluten peptide constituents of Nexvax2 each at 50 μg/ml added. The peptides corresponded to α-gliadin W02-E7, ω-gliadin/C-hordein W03-E7, and hordein B08-E2E7 reported by Tye-Din *et al*. (*7*). Peptides were at least 95% pure by high-performance liquid chromatography and liquid chromatography mass spectroscopy (CSBio, Menlo Park, California, USA). Tubes were incubated at 37°C for 24 hours before centrifugation and separation of plasma. Stimulation index was calculated by dividing cytokine concentration for Nexvax2 peptide incubation by the PBS control.

### Peripheral blood B cells

PBMC were prepared by Ficoll-Paque density centrifugation. Isolated B cells were prepared from PBMC using the Dynabeads Untouched Human B Cells Kit (11351D, Thermo Fisher Scientific) according to the manufacturer’s recommended protocol. Purity of the B cell suspension was assessed by flow cytometry, indicating a 99% CD19^+^ cell population consistent with high purity of B cells. B cells were resuspended 0.5 million/ml ml in RPMI 1640 containing 10% heat-inactivated human serum, 2 mM l-glutamine, 1 mM Na-pyruvate, 0.1 mM nonessential amino acids, 50 μM β-mercaptoethanol, streptomycin (100 μg/ml), and penicillin (100 U/ml), alone, or supplemented with IL-4 (20 ng/ml; PeproTech, Rocky Hill, NJ) plus anti-human IgA + IgG + IgM (30 μg/ml; Jackson ImmunoResearch Laboratories, West Grove, PA) plus sCD40L (1000 ng/ml; Enzo Life Sciences, Farmingdale, NY). B cells (0.1 million) were added in 0.2 ml per well in 96-well U-bottom tissue culture plates and incubated at 37°C in 5% CO_2_. After 24 hours, supernatants were harvested and frozen until evaluation.

### Cytokine assessments

Cytokines and chemokines from EDTA plasma, plasma from whole-blood IFN-γ release assay incubations, and medium from in vitro cell stimulations were measured at ImmusanT Inc. using a magnetic bead–based assay according the manufacturer’s protocol (MILLIPLEX MAP Human Cytokine/Chemokine Magnetic Bead Panel; EMD Millipore Corp., Billerica, MA, USA; MAGPIX, Luminex Corporation, Austin, TX, USA). Final concentrations were the average of triplicate measurements. Olink Proteomics (Uppsala, Sweden) was provided frozen EDTA plasma collected at baseline 2, 4, and 6 hours after the first dose from three patients in the first cohort and three in the last cohort of the 16-dose study. Olink performed a 92-plex PEA with the Proseek Multiplex Inflammation I 96 × 96 panel. Cytokine data were expressed as relative fold change from predose levels. IFN-γ, IL-1β, IL-2, IL-4, IL-6, IL-8, IL-10, IL-12p70, IL-13, TNF-α, eotaxin, eotaxin-3, IL-8, IL-8 (HA), IP-10, MCP-1, MCP-4, MDC, MIP-1α, MIP-1β, TARC (thymus and activation regulated chemokine) in EDTA plasma, and sera were measured at ImmusanT Inc. using ECL assay kits (V-PLEX Proinflammatory Panel 1 Human Kit, V-PLEX Chemokine Panel 1 Human Kit, or V-PLEX IL-2, IL-8, and IL-10 as a three-plex; Meso Scale Discovery, Rockville, MD). Data were analyzed by DISCOVERY WORKBENCH 4.0. Calculated values lower than LLOQ (lower limit of quantification) or values on the low end of the scale that were not achieved using the standard curves, were reported as equal to the LLOQ concentration analyzed for each cytokine on each plate.

### Statistical analysis

All statistical analyses were performed with GraphPad Prism version 7.0d and MathWorks MATLAB version 9.5. Nonparameteric tests were used to assess significance. All *P* values were adjusted by Benjamini-Hochberg method for multiple hypothesis testing. Cytokine signature in response to Nexvax2 administration was determined by comparing baseline-adjusted fold-change differences between placebo (*N* = 28) and active (*N* = 54) arms at specific time points after dosing to identify sequentially activated hierarchy of cytokines. *P* values were estimated by Mann-Whitney *U* test. Cytokines with average fold change of >2-fold and false discovery rate–adjusted *P* value of <0.05 were considered significant. Pearson’s correlation analysis between IL-2 concentration at 4 hours and concentrations of IL-8 and MCP-1 at 4 hours, and IP-10 at 6 hours, was used to assess strength and significance of correlation. Cytokines significant at onset of vomiting after first dose of Nexvax2 were determined by comparing fold-change response of Nexvax2-responsive signature cytokines, at onset of vomiting, in Nexvax2-treated patients (*N* = 20) with peak response observed in placebo-treated patients (*N* = 28). Concentration of each cytokine was inferred by linear interpolation at time of vomiting for each patient (*N* = 20). Peak fold-change values were used for placebo-treated patients (*N* = 28). *P* values were assessed by Mann-Whitney *U* test.

Linear mixed effects regression modeling was used to test association of vomiting with higher cytokine response. Cytokine response at 4 hours for IL-2, IL-8, MCP-1, and IP-10 were modeled as a function of nine predictor variables including age, gender, height, weight, body mass index, HLA-DQ2.5 homozygosity status, exposure to the previous gluten challenge, Nexvax2 dose levels, and occurrence of vomiting. Analysis of plasma IL-2 levels, nausea score, and occurrence of vomiting was analyzed using a logistic dose-response curve fitted to model Nausea scores (*Y*) as a function of plasma (log-transformed) IL-2 levels (*X*). The 4-parameter logistic dose-response curve was fitted with Hill slope (*H*) = 2.75, bottom (*B*) and top (*T*) nausea scores of 1 and 7, respectively, and half maximal effective concentration (EC_50_) = 62.19 pg/ml by ordinary least squares method. The model [*Y* = *B* + (T − B)/(10^((log(EC_50_) − *X*]*H))] was used to estimate the threshold of plasma IL-2 concentration (first *X* value at which *Y* > 1) beyond which significant self-reported nausea scores and occurrence of vomiting are observed.

In the randomized double-blind sham-controlled gluten food challenge study, fold-change responses in cytokines at 4 hours after gluten challenge (*N* = 11) and placebo controls (*N* = 8) were compared using Mann-Whitney *U* test. In the study investigating cytokine response to muesli bars containing 5.7 g of gluten, paired changes in cytokine concentrations at 4 hours after challenge were compared with baseline levels using Wilcoxon signed rank test. Elevation of circulating levels of IL-2, IL-8, and IL-10 after muesli challenge was summarized as area under the curve using trapezoidal numerical integration from 0 to 6 hours, and their correlation with prechallenge baseline frequency of circulating gluten-specific CD4^+^ T cells and duodenal histology measured by villous height–to–crypt depth ratio were independently assessed by Pearson’s correlation analysis.

## Supplementary Material

http://advances.sciencemag.org/cgi/content/full/5/8/eaaw7756/DC1

Download PDF

## SUPPLEMENTARY MATERIALS

Supplementary material for this article is available at http://advances.sciencemag.org/cgi/content/full/5/8/eaaw7756/DC1 Fig. S1. Activation of immune response by gluten peptides. Fig. S2. Effects of HLA genotype and previous gluten exposure on cytokine response and cytokine response stratified by Nausea scores or occurrence of vomiting. Fig. S3. Cytokine release assessed in plasma and serum. Fig. S4. Gating strategy for the generation of primary human CD4^+^ T cell lines. Fig. S5. Cytokine release in gluten-specific CD4^+^ T cell clones and short-term CD4^+^ T cell lines. Fig. S6. Cytokine release in gluten-specific CD4^+^ T cell clones, short-term CD4^+^ T cell lines, and antigen-presenting cells. Fig. S7. Cytokine release in fresh whole blood incubated with Nexvax2 peptides for 24 hours. Table S1. Plasma cytokines after intradermal Nexvax2 assessed with 38-plex magnetic bead assay. Table S2. Plasma cytokines after intradermal Nexvax2 assessed with 18-plex ECL assay. Table S3. Characteristics of patients enrolled in masked, 3-gram gluten food challenge study.
