# Supplementary material for: Cytokine release and gastrointestinal symptoms after gluten challenge in celiac disease
Source: Sci Adv. 2019 Aug 7;5(8):eaaw7756. doi: 10.1126/sciadv.aaw7756 (PMC6685723; doi:10.1126/sciadv.aaw7756)
Supplement: http://advances.sciencemag.org/cgi/content/full/5/8/eaaw7756/DC1 [file supp_5_8_eaaw7756__index.html]

Science Advances | Science AdvancesAAASSearchScience AdvancesMenu

## Supplementary Materials

**This PDF file includes:**

- Fig. S1. Activation of immune response by gluten peptides.
- Fig. S2. Effects of HLA genotype and previous gluten exposure on cytokine response and cytokine response stratified by Nausea scores or occurrence of vomiting.
- Fig. S3. Cytokine release assessed in plasma and serum.
- Fig. S4. Gating strategy for the generation of primary human CD4+ T cell lines.
- Fig. S5. Cytokine release in gluten-specific CD4+ T cell clones and short-term CD4+ T cell lines.
- Fig. S6. Cytokine release in gluten-specific CD4+ T cell clones, short-term CD4+ T cell lines, and antigen-presenting cells.
- Fig. S7. Cytokine release in fresh whole blood incubated with Nexvax2 peptides for 24 hours.
- Table S1. Plasma cytokines after intradermal Nexvax2 assessed with 38-plex magnetic bead assay.
- Table S2. Plasma cytokines after intradermal Nexvax2 assessed with 18-plex ECL assay.
- Table S3. Characteristics of patients enrolled in masked, 3-gram gluten food challenge study.

Download PDF

**Files in this Data Supplement:**

- Adobe PDF - aaw7756\_SM.pdf
