# Supplementary material for: Cytokine release and gastrointestinal symptoms after gluten challenge in celiac disease
Source: Sci Adv. 2019 Aug 7;5(8):eaaw7756. doi: 10.1126/sciadv.aaw7756 (PMC6685723; doi:10.1126/sciadv.aaw7756)
Supplement: Download PDF [file aaw7756_SM.pdf]

Science Advances
AAAS

advances.sciencemag.org/cgi/content/full/5/8/eaaw7756/DC1

Supplementary Materials for

Cytokine release and gastrointestinal symptoms after gluten challenge in celiac disease

Gautam Goel, Jason A. Tye-Din, Shuo-Wang Qiao, Amy K. Russell, Toufic Mayassi, Cezary Ciszewski, Vikas K. Sarna, Suyue Wang, Kaela E. Goldstein, John L. Dzuris, Leslie J. Williams, Ramnik J. Xavier, Knut E. A. Lundin, Bana Jabri, Ludvig M. Sollid, Robert P. Anderson*

*Corresponding author. Email: bob@immusant.com

Published 7 August 2019, *Sci. Adv.* **5**, eaaw7756 (2019)
DOI: 10.1126/sciadv.aaw7756

This PDF file includes:

Fig. S1. Activation of immune response by gluten peptides.
Fig. S2. Effects of HLA genotype and previous gluten exposure on cytokine response and cytokine response stratified by Nausea scores or occurrence of vomiting.
Fig. S3. Cytokine release assessed in plasma and serum.
Fig. S4. Gating strategy for the generation of primary human $CD4^+$ T cell lines.
Fig. S5. Cytokine release in gluten-specific $CD4^+$ T cell clones and short-term $CD4^+$ T cell lines.
Fig. S6. Cytokine release in gluten-specific $CD4^+$ T cell clones, short-term $CD4^+$ T cell lines, and antigen-presenting cells.
Fig. S7. Cytokine release in fresh whole blood incubated with Nexvax2 peptides for 24 hours.
Table S1. Plasma cytokines after intradermal Nexvax2 assessed with 38-plex magnetic bead assay.
Table S2. Plasma cytokines after intradermal Nexvax2 assessed with 18-plex ECL assay.
Table S3. Characteristics of patients enrolled in masked, 3-gram gluten food challenge study.

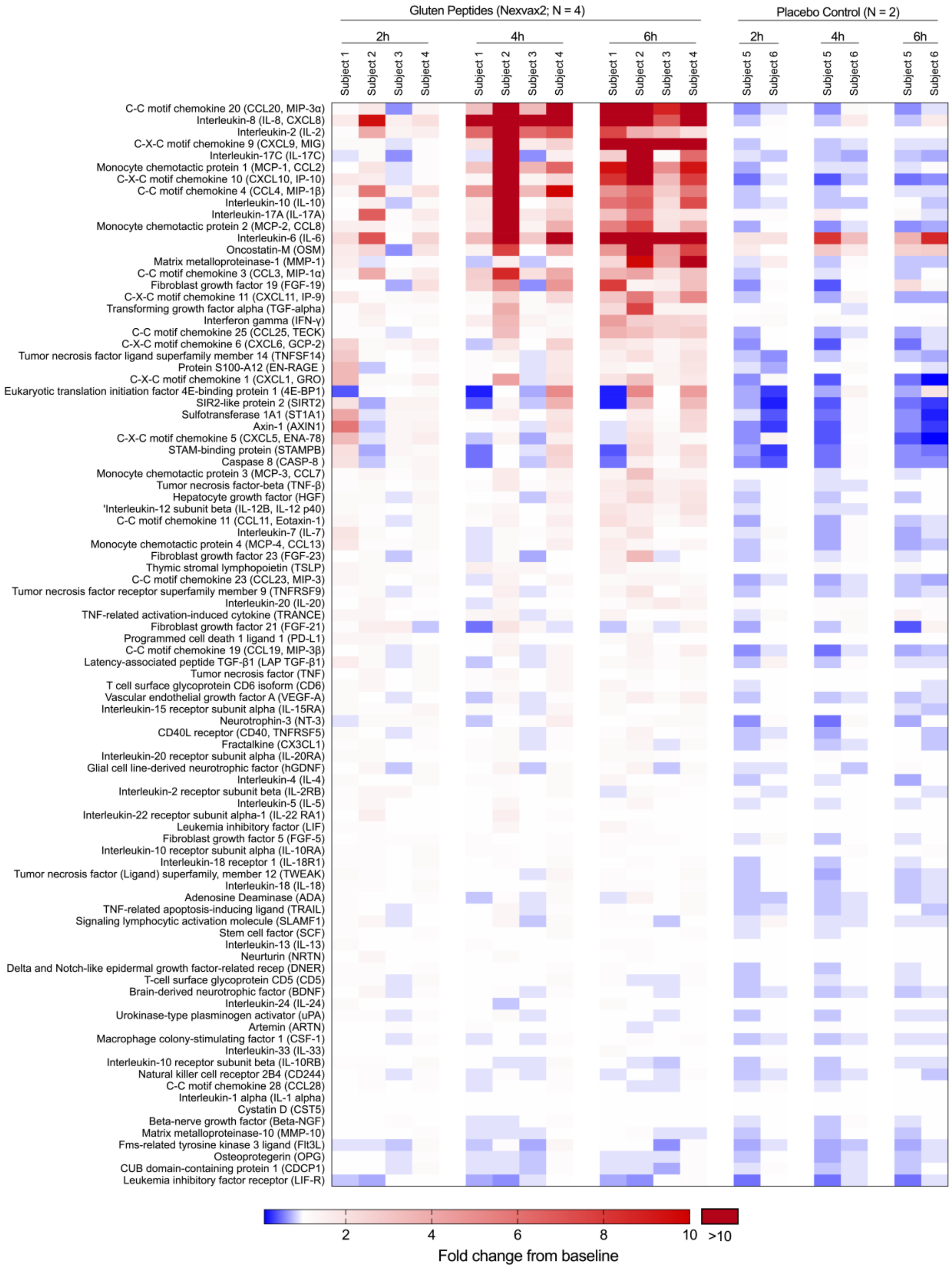

Fig. S1. Activation of immune response by gluten peptides. A 92-plex proximity extension assay was used to assess cytokines and chemokines in plasma up to 6-hours after intradermal administration of gluten peptides (Nexvax2 150 µg; N = 4) or matched placebo control (N = 2). Temporal concentration profile for each patient was transformed to a fold change response relative to individual's pre-dose baseline.

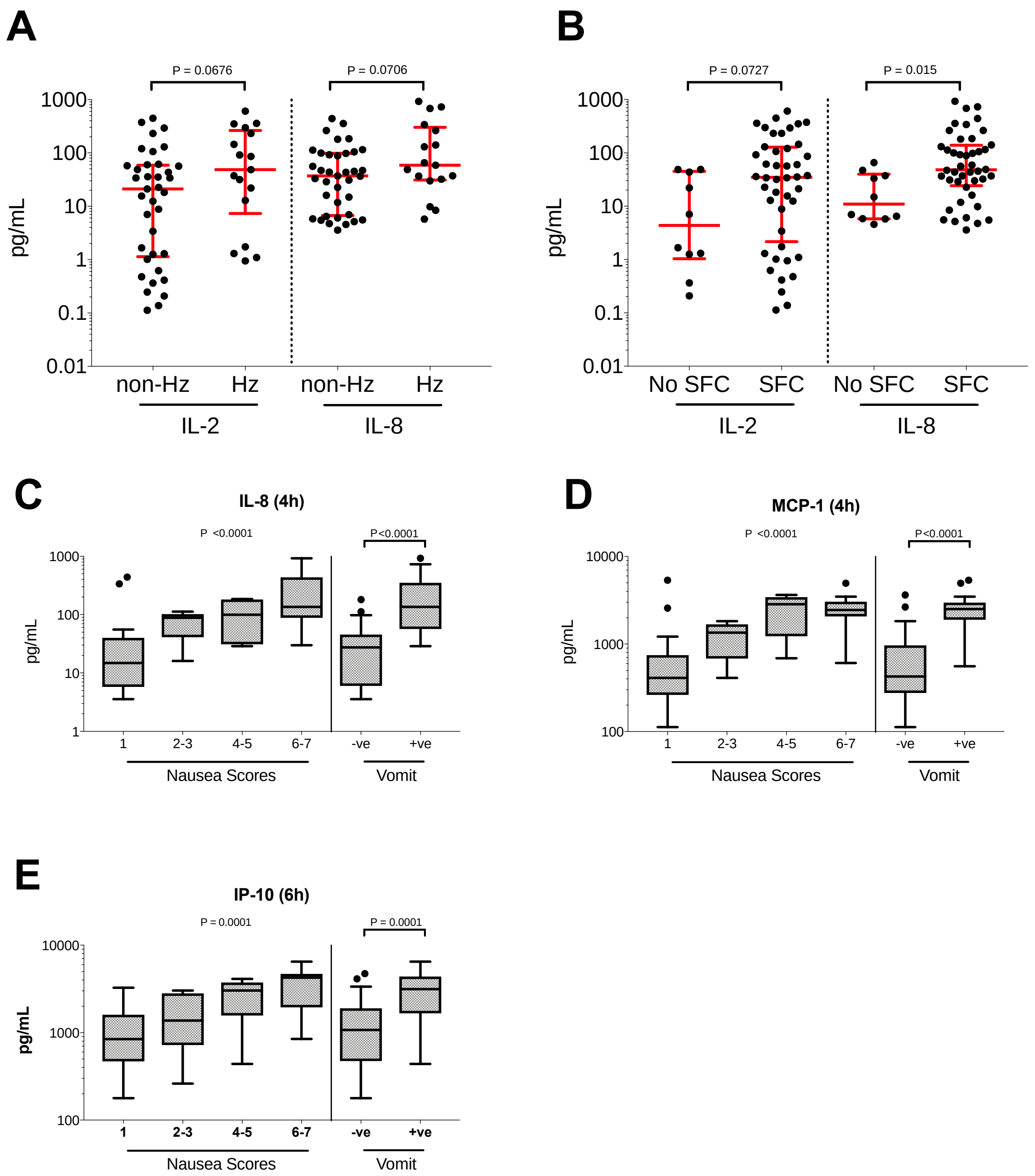

Fig. S2. Effects of HLA genotype and previous gluten exposure on cytokine response and cytokine response stratified by Nausea scores or occurrence of vomiting. (A) Plasma IL-2 concentration in CeD patients, either homozygous (Hz) or non-homozygous (non-Hz) for HLA-DQ2.5, after being treated with first dose of Nexvax2 (60, 90, 150 or 300 µg). P-values estimated using Mann-Whitney U-test. **(B)** Plasma IL-2 concentration in CeD patients who did or did not receive a screening food challenge (SFC) 4 weeks prior to first dose of Nexvax2 (60, 90, 150 or 300 µg). P-values estimated using Mann-Whitney U-test. Concentration for IL-8 at 4 hours **(C)**, MCP-1 at 4 hours **(D)**, and IP-10 at 6 hours **(E)** after first dose of Nexvax2 (60, 90, 150 or 300 µg) stratified by either increasing patient-reported Nausea score or occurrence of vomiting. Median and interquartile ranges are shown. For nausea scores, p-value was estimated by a non-parametric one-way ANOVA (Kruskal-Wallis test). Significance of difference in cytokine response between vomiters and non-vomiters was computed by Mann-Whitney U-test.

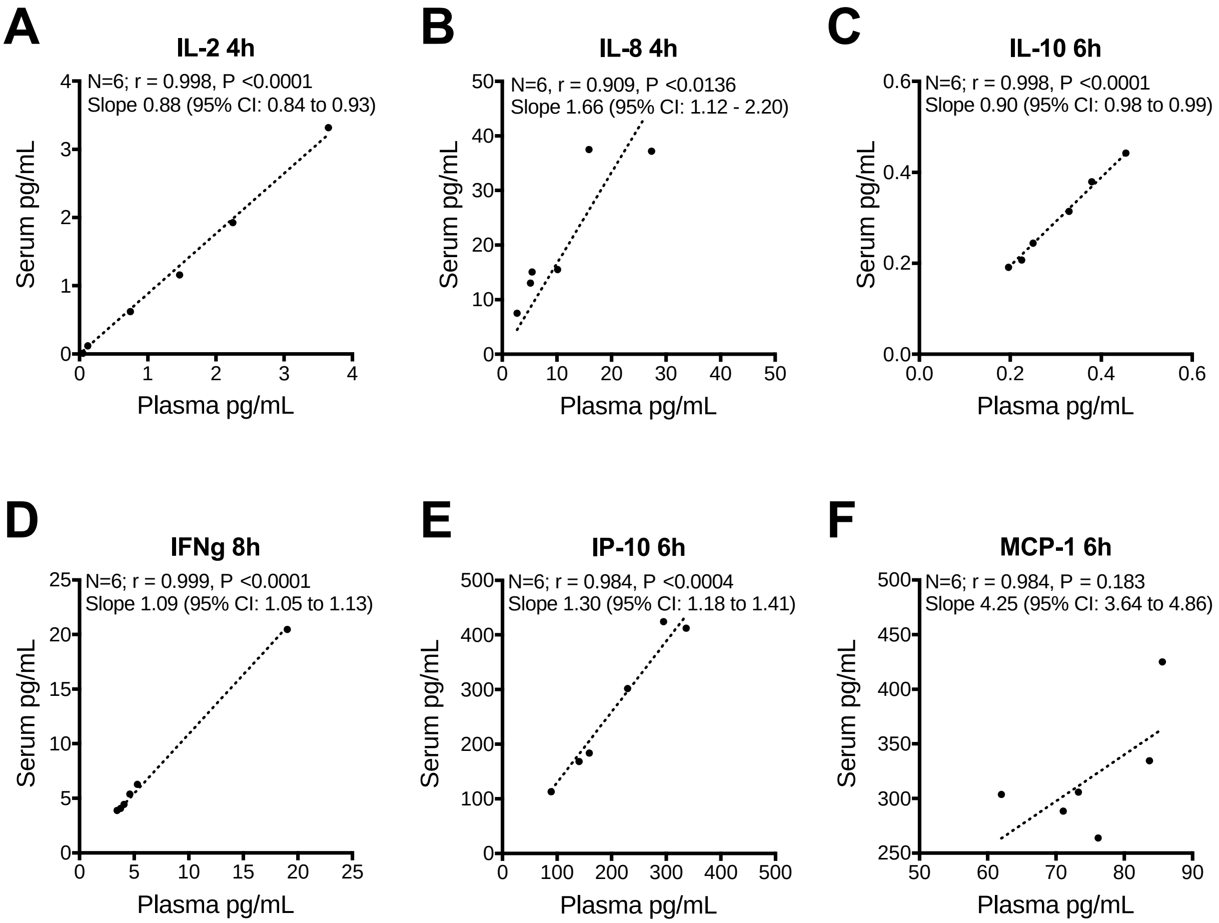

Fig. S3. Cytokine release assessed in plasma and serum. Cytokine and chemokine concentrations assessed in plasma and serum of CeD patients subject to a 3g gluten food challenge. Pearson’s correlation analysis was used to assess strength and significance of correlation.

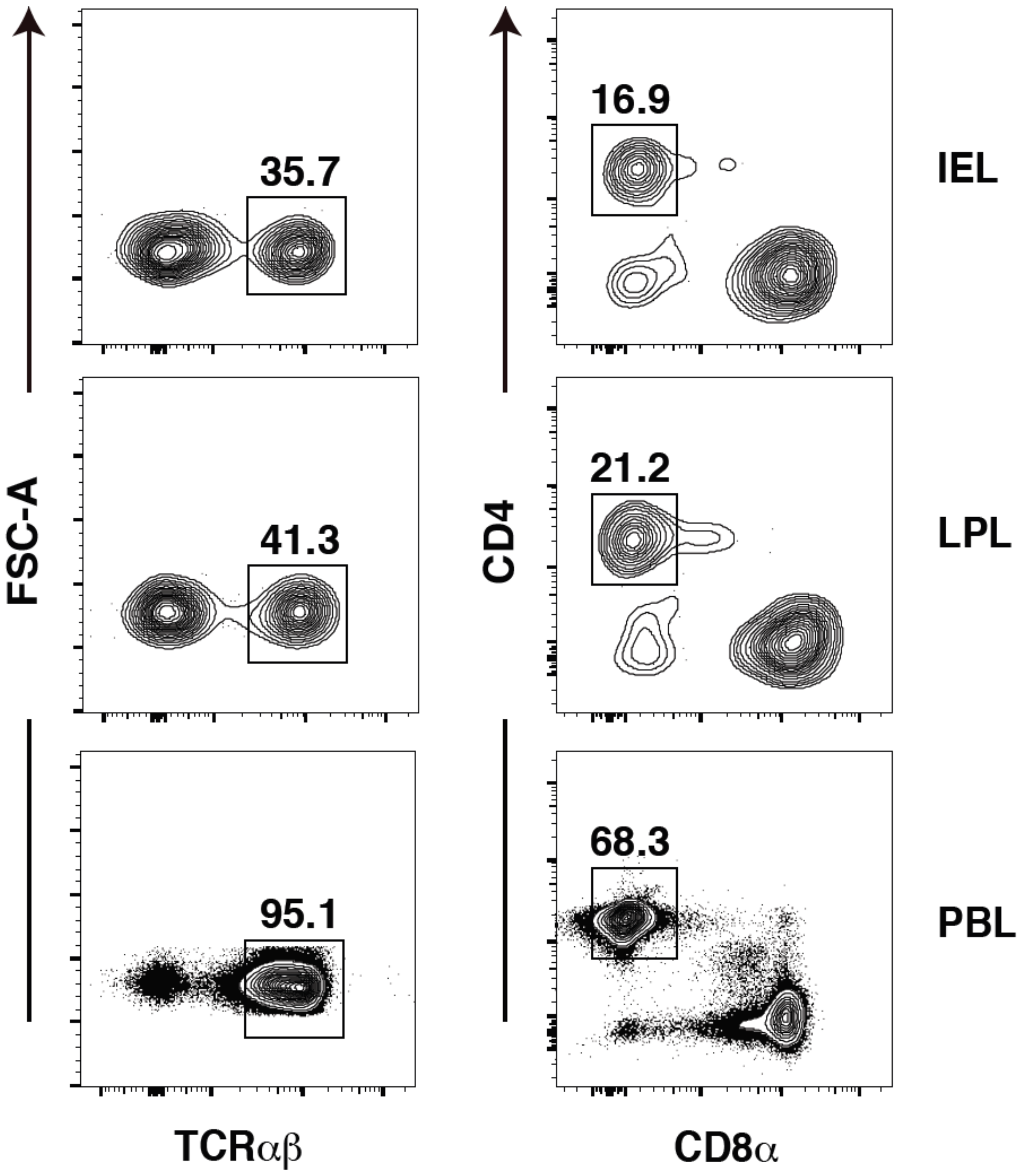

Fig. S4. Gating strategy for the generation of primary human CD4$^+$ T cell lines. Intraepithelial lymphocytes (IEL), lamina propria lymphocytes (LPL), and peripheral blood lymphocytes (PBL), were isolated and sorted based on sequential positive surface expression of TCRαβ and CD4. Cells were pre-gated on positive expression of CD3.

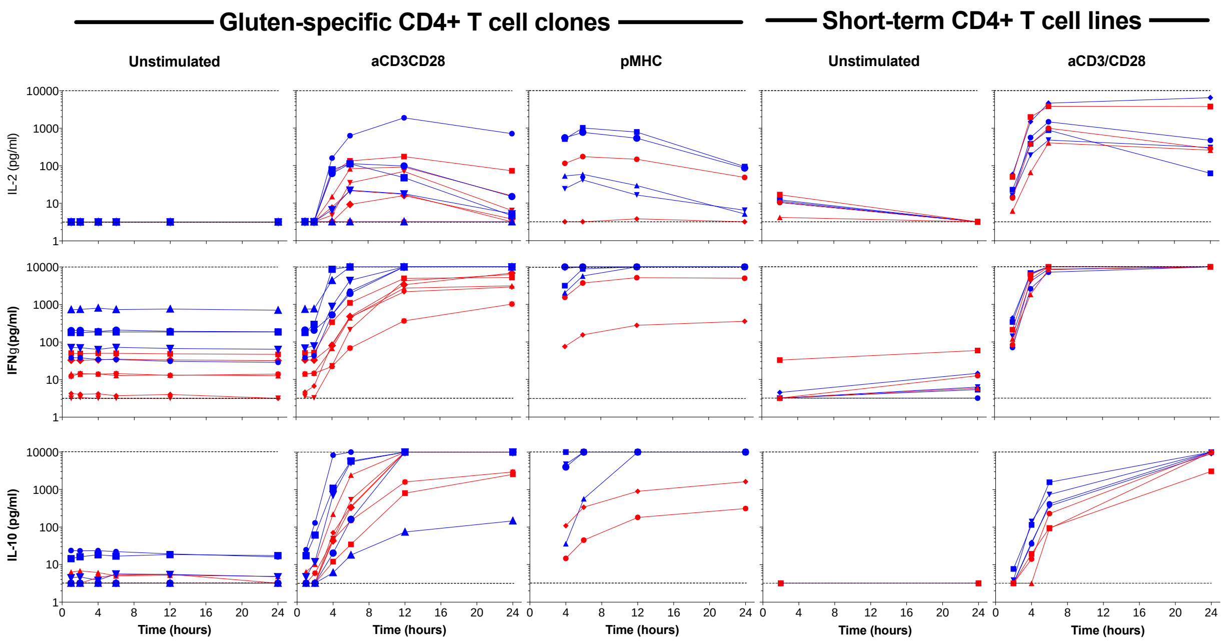

Fig. S5. Cytokine release in gluten-specific CD4$^+$ T cell clones and short-term CD4$^+$ T cell lines. Temporal cytokine release profiles of IL-2, IFN-γ and IL-10 by unstimulated or activated CD4+ T cell clones and T cell lines are shown. CD4+ T-cell clones, specific for HLA-DQ2.5-restricted epitopes in Nexvax2, were derived from either peripheral blood (red) or intestinal biopsies (blue) of CeD patients. Cytokine release was assessed after incubation with anti-CD3 and anti-CD28 antibody, or with recombinant HLA-DQ2.5 molecules bound with gluten epitope peptide (pMHC) specific for the T-cell clone, in the absence of other cell types for 24 hours. Short-term CD4+ T-cell lines were derived from either peripheral blood (red) or intestinal biopsies (blue) of CeD patients. Cytokine release was assessed after incubation with anti-CD3 and/or anti-CD28 antibody in the absence of other cell types for 24 hours.

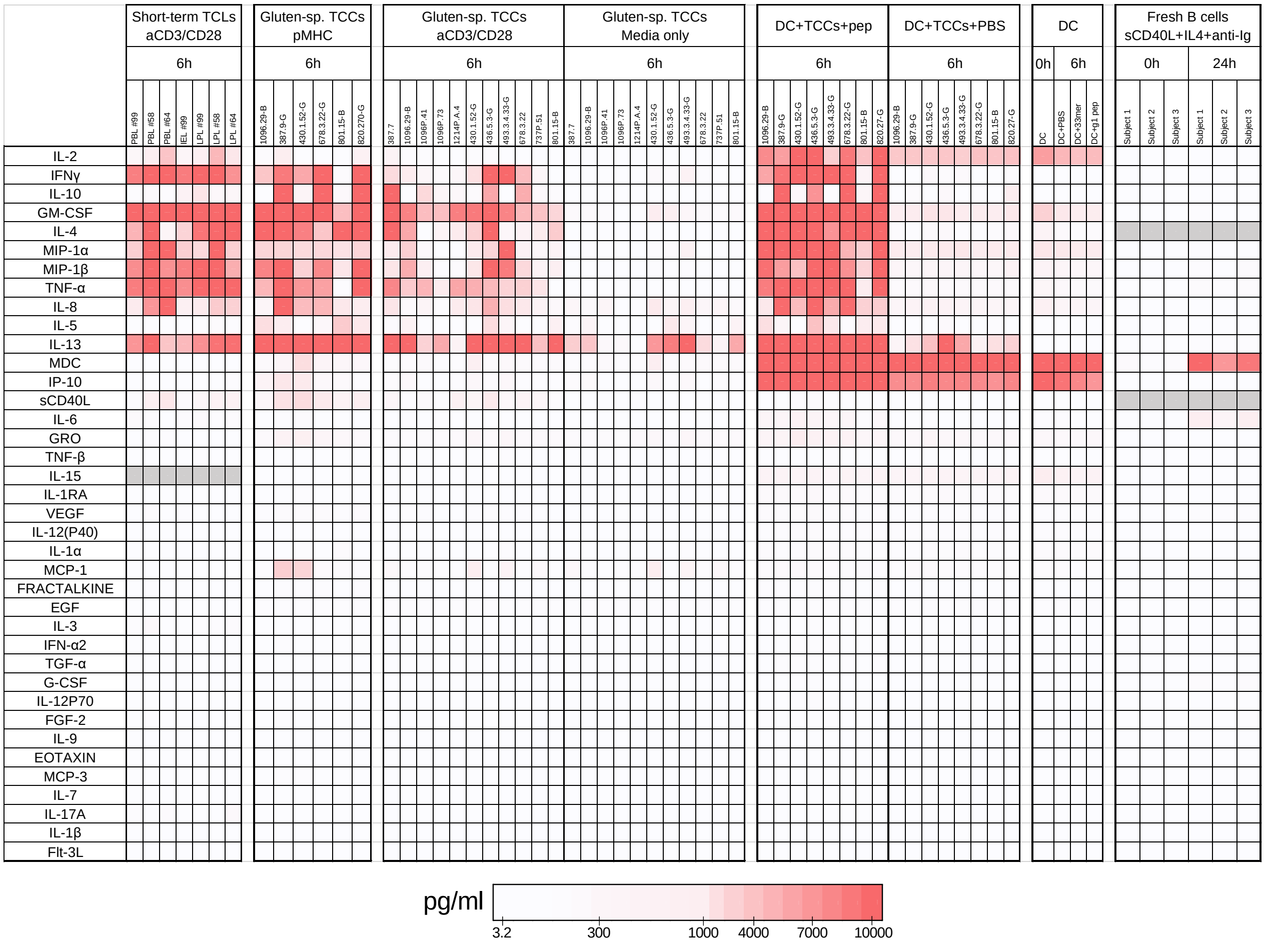

Fig. S6. Cytokine release in gluten-specific CD4$^+$ T cell clones, short-term CD4$^+$ T cell lines, and antigen-presenting cells. Heatmap shows cytokine release by unstimulated or activated CD4+ T cell clones, T cell lines, antigen presenting cells (APCs), and co-cultures of APCs and antigen-specific T cell clones. Short-term CD4+ T-cell lines were derived from either peripheral blood or intestinal biopsies of CeD patients and incubated with anti-CD3 and/or anti-CD28 antibodies. CD4+ T-cell clones, specific for HLA-DQ2.5-restricted epitopes in Nexvax2, were derived from either peripheral blood or intestinal biopsies of CeD patients and stimulated with either antiCD3/CD28 or plate-bound peptide-MHC complex. Dendritic cells, derived from one healthy HLA-DQ2.5+ donor, were incubated with combinations of gluten-specific CD4+ T cell clones and cognate gluten peptides. B cells were derived from fresh blood of healthy donors and stimulated with sCD40L, IL4 and anti-Ig.

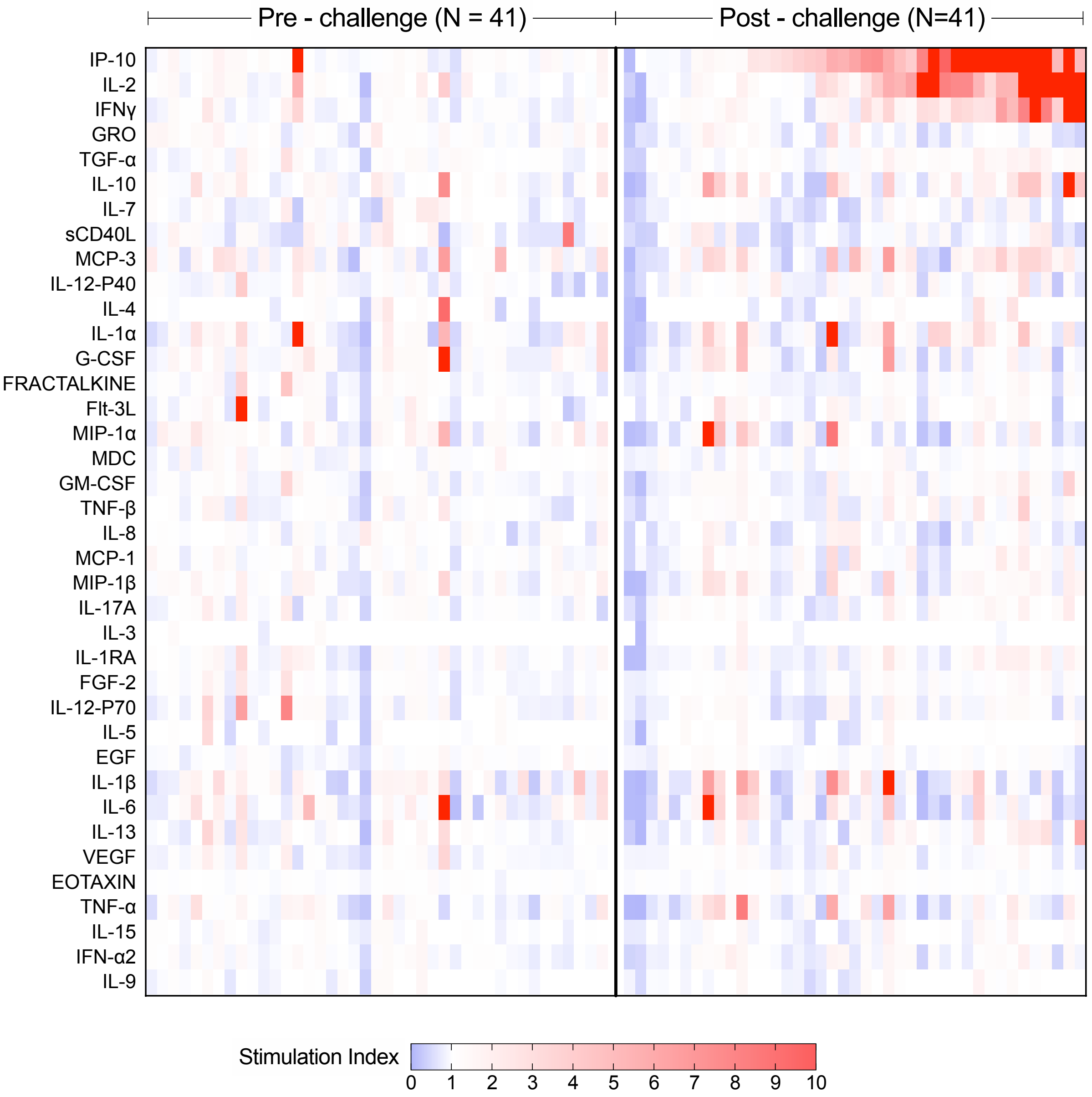

Fig. S7. Cytokine release in fresh whole blood incubated with Nexvax2 peptides for 24 hours. Whole-blood IFN-γ release assays were performed immediately before (Day 0; Pre-challenge) patients commenced the 3-day gluten challenge segment of oral gluten challenge, and 6 days later (Day 6; Post-challenge) in the screening period 4 weeks prior to receiving first dose of Nexvax2. Blood (1 mL) was collected directly into QuantiFERON-TB Gold In-Tube NIL tubes with either Nexvax2 peptides in phosphate buffered saline (PBS) or PBS only (0.1 mL). Plasma separated after 24 h incubation was frozen and later assessed for IFNγ by ELISA. All subjects randomized to treatment had a positive whole-blood IFN-γ release assay for Nexvax2 peptides in screening. Plasma samples available after the whole-blood IFN-γ release assay were analyzed by multiplex magnetic bead assay to measure a set of 38 cytokines and chemokines. Heatmap shows stimulation index, estimated as fold change observed with Nexvax2 peptide incubation relative to media only, for cytokines and chemokines from the 38plex assessed pre- and post-challenge. Statistically significant induction was observed for three cytokines, namely IL-2, IFN-γ, and IP-10.

Table S1. Plasma cytokines after intradermal Nexvax2 assessed with 38-plex magnetic bead assay. Median, IQR values are shown; BLD: below levels of detection; peak cytokine levels underlined. *: P-values assessed by Mann-Whitney U Test and corrected for multiple hypothesis testing by Benjamini-Hochberg method.

Cytokine	Function	pg/mL				Fold change from baseline			FDR Adj. P-value*		
		0h	2h	4h	6h	2h	4h	6h	2h	4h	6h
MCP-1/CCL2	Chemotactic for monocytes and basophils; mast cell release of serotonin and histamine	243(165-285)	265.9(195-316)	748(379-2376)	974(506-2624)	1.1(1.0-1.2)	3.6(1.3-9.2)	4.9(1.7-9.8)	ns	2.07E-07	2.85E-08
Interleukin-8/CXCL8	Chemotactic for neutrophils, basophils, and T cells. Neutrophil activation	11(4.6-21)	16(7.5-33)	72(28-178)	48(27-98)	1.2(1.0-1.9)	4.5(1.4-13.9)	3.9(1.7-8.4)	0.0024	6.21E-07	2.85E-08
Interleukin-2	T-cell growth and differentiation; maintain stable Treg cells in lymphoid organs and tissues	BLD(BLD-8.4)	6.7(BLD-16)	27(8.5-78)	18(5.2-35)	1.0(1.0-1.5)	3.8(1.0-13.1)	2.3(1.0-6.0)	0.0115	2.05E-06	3.53E-06
IP-10/CXCL10	Chemotactic for monocytes and T cells	440(318-716)	368(238-567)	559(383-1633)	1412(634-3036)	0.9(0.7-1.0)	1.4(0.9-2.1)	3.6(1.1-6.7)	ns	0.0003	4.56E-06
Interleukin-6	Multifunctional proinflammatory cytokine and B cells maturation factor	3.8(BLD-12)	5.7(BLD-14)	13(6.7-35)	22(11-40)	1.0(1.0-1.2)	1.5(1.0-3.7)	2.8(1.2-6.0)	ns	0.0022	5.02E-05
Interleukin-10	Reduces inflammatory cytokines secreted by macrophages and T-cells.	6.1(BLD-13)	6.1(BLD-12)	15(5.3-42)	24(8.3-39)	1.0(0.9-1.0)	1.2(1.0-3.9)	2.7(1.1-5.7)	ns	0.0024	8.48E-06
G-CSF	Granulocyte growth, differentiation and activation	34(23-66)	33(23-63)	63(28-108)	86(51-144)	0.9(0.8-1.1)	1.2(0.9-2.1)	2.3(1.1-4.1)	ns	0.0265	0.0001
MIP-1β/CCL4	Chemotactic for natural killer cells, and monocytes	25(18-50)	35(19-61)	71(35-118)	57(32-81)	1.1(0.9-1.4)	2.0(1.1-3.2)	1.9(1.2-2.6)	ns	1.10E-05	2.06E-05
Eotaxin/CCL11	Directly promotes the accumulation of eosinophils	66(52-84)	74(52-96)	108(73-181)	117(83-193)	1.0(0.9-1.2)	1.5(1.2-2.3)	2.0(1.4-2.8)	ns	2.05E-06	6.34E-08
TNF-α	Multifunctional proinflammatory cytokine	11(7-16)	14(8.6-19)	22(14-39)	21(15-31)	1.1(0.9-1.5)	1.9(1.0-3.4)	1.9(1.2-2.7)	ns	9.72E-06	1.86E-06
GM-CSF	Granulocyte, macrophage, & erythrocyte growth, differentiation and activation	19(9-37)	25(12-38)	37(21-58)	35(23-47)	1.0(0.9-1.3)	1.5(1.1-2.8)	1.7(1.1-2.5)	ns	0.0007	3.46E-05
Interleukin-1RA	Inhibits proinflammatory activity of IL-1 by binding to the IL-1 receptor	48(32-103)	55(28-105)	82(57-193)	109(65-244)	1.0(0.8-1.1)	1.5(1.1-2.2)	1.7(1.1-2.9)	ns	ns	7.42E-05
IFN-α2	Differentiation and activation of DCs	29(14-56)	26(16-58)	44(25-81)	44(30-81)	1.0(0.8-1.2)	1.3(1.0-2.2)	1.6(1.1-2.4)	ns	ns	0.0008
VEGF	Growth and migration of endothelial cells; vascular permeabilization.	150(46-433)	138(56-396)	153(81-476)	156(88-364)	1.0(0.8-1.2)	1.2(1.0-1.8)	1.2(1.0-2.0)	ns	0.0198	0.0087
Interleukin-1α	Proinflammatory, B cell, T cel, & fibroblast maturation & growth	17(BLD-85)	16(BLD-112)	22(9-109)	29(16-71)	1.0(0.8-1.0)	1.1(0.9-2.3)	1.2(1.0-4.0)	ns	ns	0.0011
IFN-γ	From antigen-activated T cells & activated NK cells.Macrophage activation.	16.1(6.2-60.0)	17.2(6.4-64.1)	21.5(9.0-64.3)	24.1(11.6-51.9)	1.0(0.8-1.2)	1.2(1.0-1.6)	1.3(1.0-1.9)	ns	ns	ns

Table S2. Plasma cytokines after intradermal Nexvax2 assessed with 18-plex ECL assay. Median, IQR values are shown; BLD: below levels of detection; peak cytokine levels underlined. *: P-values assessed by Mann-Whitney U Test and corrected for multiple hypothesis testing by Benjamini-Hochberg method.

Cytokine	Function	pg/mL				Fold change from baseline			FDR Adj. P value*		
		0h	2h	4h	6h	2h	4h	6h	2h	4h	6h
Interleukin-2	T-cell growth and differentiation; maintain stable Treg cells in lymphoid organs and tissues	0.2(0.1-0.2)	2.2(0.6-3.8)	23(3.0-52)	11(2.1-21)	14(3.5-26)	127(35-252)	70(17-231)	0.0079	0.0079	0.0079
Interleukin-8/CXCL8	Chemotactic for neutrophils, basophils, and T cells. Neutrophil activation	2.7(2.4-3.2)	4.4(3.3-5.8)	34(8.0-44)	15(10-25)	1.6(1.0-2.0)	11(3.2-12)	5.4(3.9-8.2)	ns	0.0079	0.0079
MCP-1/CCL2	Chemotactic for monocytes and basophils; mast cell release of serotonin and histamine	66(57-72)	68(61-86)	261(91-283)	336(143-414)	1.1(1.0-1.2)	3.8(1.6-4.9)	5.5(2.1-6.1)	ns	ns	0.0079
Interleukin-10	Reduces inflammatory cytokines secreted by macrophages and T-cells.	0.2(0.1-0.2)	0.2(0.2-0.2)	0.3(0.2-0.3)	0.7(0.3-1.2)	0.9(0.8-1.3)	1.7(1.0-2.3)	3.9(2.0-7.2)	ns	ns	0.0079
IFN-γ	From antigen-activated T cells & activated NK cells.Macrophage activation.	7.1(3.7-11)	6.9(3.9-11)	12(5.2-18)	26(9.6-46)	1.0(1.0-1.1)	1.5(1.2-1.8)	3.2(2.0-4.6)	ns	ns	0.0194
MIP-1β/CCL4	Chemotactic for natural killer cells, and monocytes	40(27-51)	60(34-69)	163(57-205)	130(64-137)	1.3(1.1-1.6)	3.2(1.7-3.4)	2.6(1.9-3.1)	ns	0.0114	0.0315
IP-10/CXCL10	Chemotactic for monocytes and T cells	256(149-319)	212(130-221)	232(139-468)	441(255-1243)	0.8(0.7-0.9)	1.1(0.9-1.2)	2.7(1.4-3.1)	ns	ns	0.0079
Eotaxin/CCL11	Directly promotes the accumulation of eosinophils	79(68-88)	82(71-103)	114(103-149)	156(108-200)	1.1(0.9-1.4)	1.7(1.2-2.0)	2.2(1.4-2.5)	ns	ns	0.0194
TNF-α	Multifunctional proinflammatory cytokine	1.9(1.7-2.0)	2.1(1.7-2.5)	4.3(2.2-4.9)	3.9(2.5-4.4)	1.1(1.0-1.1)	2.0(1.2-2.4)	2.0(1.4-2.1)	ns	0.0114	ns

Table S3. Characteristics of patients enrolled in masked, 3-gram gluten food challenge study. GFD gluten-free diet; CDAT Celiac Dietary Adherence Test; *Elevated above upper level of normal; # S serum P EDTA plasma

Subject	Gender	Age/yr	GFD duration/yr	CDAT	HLA-DQ genotype	TG2 IgA serology	DGP IgG serology	Food challenge	Blood collections/h	Blood samples#
0564	F	70	10	10	2.5/X	1	3	Gluten	0,1,2,3,4,5,6,7,8	S+P
0577	F	45	5	8	2.5/2.5	<1	1	Gluten	0,1,2,3,4,5,6,7,8	S+P
0211	M	65	18	7	2.5/X	2	2	Gluten	0,1,2,3,4,5,6,7,8	S+P
0945	M	62	5	9	2.5/X	<1	1	Gluten	0,1,2,3,4,5,6,7,8	S+P
0537	F	36	21	12	2.5/2.5	1	1	Gluten	0,1,2,3,4,5,6,7,8	S+P
0974	M	24	7.5	11	2.5/8	<1	2	Gluten	0,4,6	S
0907	F	24	4	11	2.5/X	<1	2	Gluten	0,4,6	S
0309	F	60	12	8	2.5/8	<1	1	Gluten	0,4,6	S
1143	F	49	10	10	2.5/X	<1	1	Gluten	0,4,6	S
1169	F	51	1.5	7	2.5/8	<1	22*	Gluten	0,4,6	S
0043	F	65	12	11	2.5/X	<1	<1	Gluten	0,4,6	S
0240	F	58	16	7	2.5/X	<1	6	Gluten-free	0,1,2,3,4,5,6,7,8	S+P
1157	F	21	6	9	2.5/X	<1	2	Gluten-free	0,4,6	S
0573	M	60	11	9	2.5/X	<1	1	Gluten-free	0,4,6	S
0650	F	58	5	7	2.5/X	1	3	Gluten-free	0,4,6	S
0192	M	63	18	7	2.5/X	<1	2	Gluten-free	0,4,6	S
0188	F	66	15	10	2.5/X	<1	5	Gluten-free	0,4,6	S
3010	M	20	5	9	2.5/2.5	<1	25*	Gluten-free	0,4,6	S
0648	F	54	6	8	2.5/2.5	<1	4	Gluten-free	0,4,6	S
Median (IQR)		58 (36-63)	10 (5-15)	9 (7-10)						
Mean (SD)		50 (17)	10 (6)	9 (2)						
